# Birth weight, ototoxic medication, and surgical history predict individual hearing loss risks: a systematic review and meta-analysis

**DOI:** 10.3389/fped.2026.1729458

**Published:** 2026-02-06

**Authors:** Hanwen Luo, Jianghua He, Dapeng Chen, Xiaoming Xu, Jing Zhao, Xiaoyan Yang, Jing Shi

**Affiliations:** 1 Department of Pediatrics, West China Second University Hospital, Chengdu, Sichuan, China; 2 Key Laboratory of Birth Defects and Related Diseases of Women and Children, Ministry of Education, Sichuan University, Chengdu, Sichuan, China

**Keywords:** HL, newborns, NICU, ototoxic medication, surgical history

## Abstract

**Background:**

Hearing loss (HL) impairs sound perception and includes sensorineural, conductive, and mixed subtypes. Compared with healthy newborns, infants admitted to the neonatal intensive care unit (NICU) are at substantially increased risk of congenital anomalies and exposure to HL-related risk factors. However, the specific determinants of neonatal HL remain controversial.

**Objective:**

This systematic review and meta-analysis seeks to identify risk factors linked to HL in neonates admitted to the NICU.

**Methods:**

PubMed, the Cochrane Library, Embase, and Web of Science were systematically searched from March 26, 1996, to February 25, 2025. Eligible studies were English-language retrospective studies employing multivariate logistic regression to evaluate potential risk factors for HL in NICU neonates. Meta-analyses were conducted using STATA, and pooled estimates were reported as odds ratios or relative risks (OR/RR) with 95% confidence intervals (CI).

**Results:**

This study included 21 retrospective studies with a total of 21,143 participants. Meta-analysis indicated that exposure to ototoxic drugs (aminoglycosides, loop diuretics), history of the surgical ligation of patent ductus arteriosus (PDA), craniofacial anomalies, family history of HL, and TORCH infections were significantly associated with HL in NICU neonates (all *P* ≤ 0.05). In contrast, low Apgar score, prematurity, low birth weight, duration of vancomycin exposure, sex, and sepsis were not significantly correlated. The findings were robust, and no evident publication bias was detected.

**Conclusion:**

Statistically significant risk factors for HL included craniofacial anomalies, family history of HL, TORCH infections, and surgical ligation of PDA, as well as hyperbilirubinemia, exposure to loop diuretics and other ototoxic drugs, and mechanical ventilation (all *P* ≤ 0.05). In contrast, low Apgar score, preterm birth, and sepsis were not significantly linked to HL.

**Systematic Review Registration:**

https://www.crd.york.ac.uk/PROSPERO/search, PROSPERO CRD420250653476.

## 1 Introduction

Hearing loss (HL), including sensorineural hearing loss (SNHL), conductive hearing loss (CHL), and mixed hearing loss (MHL), is a congenital or perinatal condition that can profoundly impair language acquisition, cognitive development, and social integration in neonates. Infants admitted to neonatal intensive care units (NICUs) are at substantially increased risk of hearing impairment due to exposure to multiple adverse medical conditions and intensive interventions. Epidemiological evidence indicates that the incidence of SNHL among NICU neonates ranges from 1% to 10%, which is approximately 30-fold higher than the 0.1% prevalence observed in the general newborn population (1, 2). Furthermore, previous studies have reported that 26.3% of affected NICU neonates present with SNHL, 30.4% with CHL, and 20% with MHL (1–3). SNHL in this population is commonly attributed to immature cochlear development and environmental stressors, whereas CHL is more often linked to congenital structural anomalies or acquired inflammatory conditions (4). These alarming statistics underscore the critical need to systematically identify and validate key HL risk factors in NICU neonates.

Otoacoustic emissions (OAE) and auditory brainstem response (ABR) are mainstream tools for neonatal HL screening (3, 5, 6): OAE assesses cochlear function with high sensitivity (7), while ABR evaluates central auditory pathway integrity and behavioral thresholds. Per JCIH recommendations, a two-stage screening protocol, initial OAE followed by ABR for infants who fail OAE, is widely implemented in NICUs and well-baby nurseries. OAE is preferred as a first-line screening tool for its simplicity, cost-effectiveness, and time efficiency (8). The OAE-ABR combination is regarded as the gold standard for NICU hearing screening, enabling the detection of auditory neuropathy spectrum disorders (9) and highlighting the necessity of early HL detection and intervention.

Over the past decades, the JCIH has proposed 10 major risk factors for neonatal HL, including very low birth weight (<1,500 g), exposure to ototoxic medications, and mechanical ventilation for more than five days [Joint Committee on Infant Hearing (JCIH), 1994 Position Statement] (10). However, subsequent studies have yielded inconsistent results regarding the predictive value of these factors. For example, Wang et al. (11)and Lima et al. (12) confirmed a significant association between low birth weight and SNHL, while Abdullah et al. (13) found no such correlation. Arslan et al. (14) identified ototoxic drug exposure as a strong predictor of HL, while Alaee et al. (15) and Hille et al. (16) reported contradictory findings. With respect to surgical history, ductus arteriosus ligation, a common NICU procedure, has been suggested to increase the risk of central nervous system-related hearing impairment in extremely preterm infants (5), but evidence remains limited, and large-scale validation is lacking.

Notably, several critical gaps persist in the existing literature. Most studies have analyzed NICU neonates together with community-born newborns, potentially obscuring NICU-specific risk profiles. Moreover, no meta-analysis to date has systematically evaluated the associations between core risk factors, like birth weight, ototoxic medication exposure, and surgical history, and HL specifically in NICU populations. Therefore, this systematic review and meta-analysis aim to synthesize evidence from retrospective studies using multivariate logistic regression to quantify the strength of associations between these risk factors and HL in NICU neonates. By providing robust pooled estimates, this study seeks to facilitate early identification of high-risk infants and inform targeted screening and preventive strategies.

## 2 Materials & methods

The findings of this paper were presented under the Preferred Reporting Items for Systematic Reviews and Meta-Analyses (PRISMA) statement (17) and the Cochrane Handbook for Systematic Reviews of Interventions (18). The study protocol was registered with PROSPERO (CRD420250653476).

### 2.1 Eligibility criteria

#### 2.1.1 Study type

Retrospective studies published in English between March 26, 1996, and February 25, 2025, were eligible. Included studies were required to employ multivariate logistic regression analyses to investigate risk factors for HL in NICU neonates and to report effect estimates as odds ratios (ORs) or relative risks (RRs) with corresponding 95% confidence intervals (CIs).

#### 2.1.2 Study subjects

Inclusion criteria were: (1) neonates admitted to the NICU within 28 days after birth; (2) HL diagnosed within 3 months after birth using OAE or ABR (defined as failure of OAE twice or ABR once); (3) patient characteristics and candidate risk factors, as detailed in Table 1, were reported; (4) multivariate logistic regression was performed with complete statistical results available.

**Table 1:** Included risk factors Newcastle-Ottawa scale.

Firstauthor (year)	Newcastle-Ottawa scale								
	Is the case definition adequate?	Representativeness of the cases	Selection of Controls	Definition of Controls	Comparability	Ascertainment of exposure	Same method of ascertainment for cases and controls	Non-response rate	Total score
Smith et al. (20)	★	★	★	✩	★★	★	★	✩	7
Maharani etal. (21)	★	★	✩	✩	★★	★	★	✩	6
Wang et al. (17)	★	★	★	✩	★★	★	★	★	8
Khairy et al. (9)	★	★	★	✩	★★	★	★	✩	7
Abdullah et al. (13)	★	★	★	✩	★★	★	★	✩	7
Hajare and Mudhol (22)	★	★	★	✩	★★	★	★	★	8
Van Dommelen et al. (23)	★	★	✩	✩	★★	★	★	✩	6
Lima et al. (12)	★	★	★	✩	★★	★	★	★	8
Bhat et al. (24)	★	★	★	✩	★★	★	★	★	8
Gupta et al. (25)	★	★	✩	✩	★★	★	★	✩	6
Alaee et al. (27)	★	★	★	✩	★★	★	★	✩	7
de Hoog et al. (26)	★	★	✩	✩	★★	★	★	✩	6
Robertson et al. (11)	★	★	★	✩	★★	★	★	✩	7
Cooper et al. (28)	★	★	★	✩	★★	★	★	✩	7
Eras et al. (29)	★	★	★	✩	★★	★	★	★	8
Hille et al. (15)	★	★	★	✩	★★	★	★	★	8
Leslie et al. (30)	★	★	★	✩	★★	★	★	✩	7
Arslan et al. (14)	★	★	✩	✩	★★	★	★	✩	6
Chant et al. (31)	★	★	★	✩	★★	★	★	✩	7
Wang et al. (16)	★	★	★	✩	★★	★	★	✩	7
Nair et al. (32)	★	★	★	✩	★★	★	★	★	8

#### 2.1.3 Definition of exposure and control

Exclusion criteria were: (1) animal studies; (2) duplicate publications, studies with inaccessible data, or those unsuitable for meta-analysis; (3) reviews, expert commentaries, guidelines, or opinion articles; (4) incomplete full-text availability; (5) non-English publications.

The exposure group consisted of NICU neonates diagnosed with HL who were exposed to one or more target risk factors, including ototoxic medications, low birth weight, surgical history (surgical procedures may increase HL risk through combined effects of anesthetic neurotoxicity, perioperative cerebral hemodynamic alterations, and congenital organ malformations (19), sex (sex hormone regulation, genetic architecture differentiation, and cochlear structural-functional specialization account for variations in hearing impairment), and other potential factors.

The control group included NICU neonates without exposure to the specified risk factors and without a diagnosis of HL, with baseline characteristics comparable between groups.

### 2.2 Literature search strategy

PubMed, Cochrane Library, Embase, and Web of Science were retrieved from March 26, 1996, to February 25, 2025. Medical Subject Headings (MeSH) terms were combined with free-text keywords, and the detailed search strategy is provided in Supplementary File S1. Core search terms included “newborns,” “HL,” “NICU,” “ototoxic medication,” and “surgical history,” ensuring comprehensive identification of relevant studies.

### 2.3 Data extraction and quality assessment

#### 2.3.1 Data extraction

Data were independently extracted by two investigators (Dapeng Chen and Jianghua He). Extracted information included study characteristics (first author, publication year, country, sample size, and follow-up duration), participant characteristics (gestational age and birth weight), types of risk factors, statistical models, and main outcomes (OR/RR, 95% CI, and *P* values). Discrepancies were resolved through discussion with a third reviewer (Jing Shi).

#### 2.3.2 Risk of bias assessment

The risk of bias in the included studies was assessed via the Newcastle-Ottawa Scale (NOS), which evaluates eight domains: (1) adequacy of case definition; (2) representativeness of cases; (3) selection of controls; (4) definition of controls; (5) comparability between groups; (6) ascertainment of exposure; (7) consistency of exposure assessment between cases and controls; and (8) adequacy of the non-response rate. Assessments were conducted independently by two reviewers, with disagreements resolved by consensus. Quality assessment results are shown in Table 1.

To minimize subjectivity in quality appraisal, two additional measures were implemented. First, a standardized operational guideline for judgment was established to reduce inter-reviewer variability. Second, sensitivity analyses were performed to compare pooled estimates derived from high-quality studies with those from all included studies, thereby evaluating the robustness of the conclusions against potential bias.

### 2.4 Statistical analysis

STATA was used for data analysis, with pooled effect sizes presented as odds ratios (OR) or relative risk (RR) and 95% confidence interval (CI). Heterogeneity between studies was first assessed using the *I*^2^ statistic and *P*-value: if *P* < 0.05 and/or *I*^2^ > 50%, significant heterogeneity was indicated, and the DerSimonian-Laird random-effects model was used for data pooling [weight *W_i_* = 1/(*V_i_* + *τ*^2^), where *V_i_* is the within-study variance and *τ*^2^ is the between-study variance estimated by the restricted maximum likelihood method]; otherwise, the fixed-effects model was adopted. Sensitivity analyses were conducted by sequentially omitting individual studies to assess the stability of pooled results. Publication bias was evaluated using funnel plots and Begg's test, and the trim-and-fill method was applied when bias was detected.

### 2.5 Outcome measures

1. *P*-value;
2. OR/RR and 95% CI.

## 3 Results

**Figure 1:**
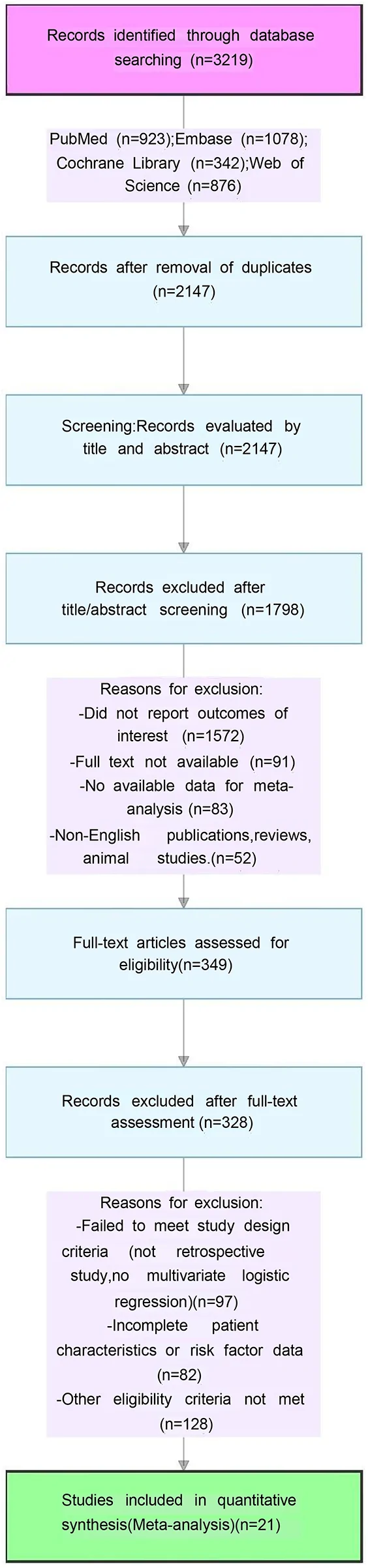
PRISMA flow diagram of the study process. PRISMA, preferred reporting items for systematic review and meta-analysis.

3,219 records were identified from PubMed (*n* = 923), Embase (*n* = 1,078), the Cochrane Library (*n* = 342), and Web of Science (*n* = 876). After removal of duplicates, 2,147 records underwent title and abstract screening, of which 1,798 were excluded (1,572 for irrelevant outcomes, 91 for unavailable full text, 83 for insufficient data for meta-analysis, and 52 for non-English publications, reviews, or animal studies). The remaining 349 articles were assessed in full text, leading to the exclusion of 328 studies (97 for inappropriate study design, 82 for incomplete patient or risk factor data, and 128 for failure to meet other eligibility criteria). Ultimately, 21 observational studies were included in the meta-analysis (Figure 1). [Smith et al. (20); Maharani et al. (21); Wang et al. (11); Khairy et al. (9); Abdullah et al. (13); Hajare et al. (22); Dommelen et al. (23); Lima et al. (12); Bhat et al. (24); Gupta et al. (25); Alaee et al. (15); Hoog et al. (26); Robertson et al. (27); Cooper et al. (28); Eras et al. (29); Hille et al. (16); Leslie et al. (30); Arslan et al. (14); Chant et al. (31); Wang et al. (11); Nair et al. (32)] were included. Characteristics of these studies are listed in Table 2.

**Table 2:** Information on included studies.

First author	Year	Country	Study design	Sample size	Follow-up	Statistical Model	Gestational age	Birth weight	Risk factors
RichardJ. H.Smith	1992	America	retrospective study	238	6-year period beginning in 1981	Stepwise Logistic Regression	NS	NS	Family history
									Craniofacial anomalies/Congenital head and neck deformity
									Low Apgar scores
									Hyperbilirubinemia
									Ototoxic medication
									Convulsions/Seizure disorder
Ni Luh Putu Maharani	2015	Bali	retrospective study	54	From November 2012 to February 2013	Multivariable logistic regression	NS	2,732 ± 7.365 g	Hyperbilirubinemia
									Ototoxic medication
									Meningitis
Chien-Ho Wang	2017	China Taiwan	retrospective study	309	From January 2010 to 2011.	Logistic Regression Analysis	27.5 ± 1.4	1,028 ± 180 g	Low birth weight
									Craniofacial anomalies/Congenital head and neck deformity
									Ototoxic medication
									Intracranial hemorrhage/Intracranial complication
									Prematurity/Shorter gestational length
									PDA surgical ligation
									Oxygen duration
May Ahmed Khairy	2018	Egypt	retrospective study	260	From May	Logistic regression	32.7 weeks	1,750 gm	Mechanical ventilation
					From May 2013 to January 2014				
Asma ABDULLAH	2020	Malaysia	retrospective study	2,713	From January 2014 to December 2016	Logistic regression	NS	NS	Low birth weight
									Craniofacial anomalies/Congenital head and neck deformity
									Mechanical ventilation
									Low Apgar scores
									Hyperbilirubinemia
									Ototoxic medication
Priti Hajare	2021	India	retrospective study	402	N	Logistic regression	NS	NS	low birth weight
									Family history
									Craniofacial anomalies/Congenital head and neck deformity
									Parental consanguinity
									Mechanical ventilation
									Hyperbilirubinemia
									Sex (male)
									Intracranial hemorrhage/Intracranial complication
									Prematurity/Shorter gestational length
									Torch infection
van Dommelen	2010	Netherlands	retrospective study	10,830	For 2002–2005	Multivariate logistic regression	NS	NS	Low birth weight
									Craniofacial anomalies/Congenital head and neck deformity
									Mechanical ventilation
									Low Apgar scores
									Sex (male)
									Sepsis
									Phototherapy
									Torch infection
									Meningitis
									Chromosomal abnormalities/Positive genetic syndrome/Down syndrome
									Oxygen duration
Gisele M.L.Lima	2006	Brasil	retrospective study	979	2000.1–2003.1	Multiple analyses by logistic regression	NS	NS	low birth weight
									Family history
									Craniofacial anomalies/Congenital head and neck deformity
									Mechanical ventilation
									Hyperbilirubinemia
									Chromosomal abnormalities/Positive genetic syndrome/Down syndrome
									Postnatal hypoxia/severe birth asphyxia
Jehangir Allam Bhat	2018	Indian	retrospective study	195	From June 2015 to May 2016	Multivariate logistic regression	NS	NS	Family history
									Low Apgar scores
									Hyperbilirubinemia
									Chromosomal abnormalities/Positive genetic syndrome/Down syndrome
A.K. Gupta	1991	India	retrospective study	24	From March 1989 to July 1989	Multiple Logistic regression analysis.	NS	NS	Low birth weight
									Hyperbilirubinemia
									Prematurity/Shorter gestational length
Ehsan Alaee	2015	Iran	retrospective study	791	2010–2011	Binary logistic regression	NS	NS	low birth weight
									Hyperbilirubinemia
									aminoglycosides
									Vancomycin
									Vancomycin duration
									Prematurity/Shorter gestational length
MATTHIJS DE HOOG	2003	Netherlands.	retrospective study	625	From November 1998 to November 2000	Multiple logistic regression	NS	NS	aminoglycosides
									Vancomycin
									Vancomycin duration
									Loop diuretics/Duration of diuretic use
CHARLENE M. T. ROBERTSON	2005	Canada	retrospective study	43	Born January 1994 through	Multivariate forward stepwise logistic regression	NS	NS	aminoglycosides
									Loop diuretics/Duration of diuretic use
Aaron C. Cooper	2011	USA	retrospective study	29	Between February 2003 and January 2008	Multivariate logistic regression	NS	NS	aminoglycosides
Zeynep Eras	2014	Turkey	retrospective study	1,360	Between September 2009 and December 2011	Multinomial logistic regression analysis	29.7 ± 2.3 weeks	1,317 ± 349.7 g	Mechanical ventilation
									PDA surgical ligation
									Loop diuretics/Duration of diuretic use
Elyśee TM Hille	2007	Netherlands	retrospective study	2,186	Between October 1, 1998, and January 1, 2002	Multivariate analysis	28.5 ± 1.6	1,039 ± 256	Mechanical ventilation
									Postnatal hypoxia/severe birth asphyxia
GI LESLIE	1995	Australia	retrospective study	102	From July 1985 through December 1990	Logistic regression analysis	26 ± 1.2	982 ± 123	Oxygen duration
S. Arslan	2013	Turkey	retrospective study	136	Between May 2007 and January 2008	Logistic regression analysis	NS	NS	Parental consanguinity
									Prematurity/Shorter gestational length
Kathy Chant	2022	UK	retrospective study	36	Between 2009 and 2013	Multiple regressions	NS	NS	sex
									Birth weight
									Low Apgar scores
Laura A. Wang	2018	USA	retrospective study	1,020	Between 2004 and 2013	Logistic regression model	NS	NS	Loop diuretics/Duration of diuretic use
Vrinda Nair	2021	England	retrospective study	122	2005–2019	Binary logistic regression	NS	NS	Craniofacial anomalies/Congenital head and neck deformity
									Mechanical ventilation
									Hyperbilirubinemia
									aminoglycosides
									Vancomycin
									Sepsis
									Convulsions/Seizure disorder
									Meningitis
Total	—	—	—	21,143	—	—	28.0 ± 6.1	1,475 ± 274 g	—

Quality assessment results are displayed in Supplementary Table S1.

### 3.1 Association between ototoxic medications and HL

Five studies examined the association between exposure to ototoxic medications and HL in neonates admitted to the NICU. Meta-analysis demonstrated a significant association between ototoxic medication exposure and increased HL risk (OR = 2.00, 95% CI: 1.52–2.63, *I*^2^ = 65.2%, *P* < 0.001) (Supplementary Figure S1).

Aminoglycosides: Data from four studies demonstrated a significant link to HL [OR = 1.95, 95% CI (1.35, 2.82), *I*^2^ = 74.8%, *P* < 0.01] (Supplementary Figure S13).

Loop diuretics: Two studies confirmed that loop diuretic use was related to an elevated risk of HL [OR = 3.26, 95% CI (2.04, 5.14), *I*^2^ = 50.0%, *P* = 0.00] (Supplementary Figure S6).

Vancomycin: Analysis of two studies suggested a potential relation of vancomycin exposure to HL [OR = 1.65, 95% CI (0.97, 2.82), *I*^2^ = 84.9%, *P* = 0.06]. No significant association was found across vancomycin treatment duration and HL [OR = 1.32, 95% CI (0.89, 1.96), *I*^2^ = 78.4%, *P* = 0.17] (Supplementary Figures S19 and S20).

### 3.2 Association between birth weight and HL

Eight studies explored the impact of low birth weight on HL in NICU neonates. Overall meta-analysis showed a potential association between low birth weight and HL [OR = 1.31, 95% CI (0.99, 1.74), *I*^2^ = 65.6%, *P* = 0.06]. Stratified analysis by birth weight category revealed:

Very low birth weight (<1,500 g): Data from 4 studies indicated no significant risk of HL in this weight group (OR = 3.05, 95%CI [0.57, 16.38], *I*^2^ = 72.8%, *P* = 0.19).

Low birth weight (1,500 g–2,500 g): Two studies showed an insignificant association between this weight range and HL [OR = 1.29, 95% CI (0.79, 2.13), *I*^2^ = 15.0%, *P* = 0.31) (Supplementary Figures S15–S17).

### 3.3 Association between surgical history and HL

Two studies evaluated the association between patent ductus arteriosus (PDA) surgical ligation and HL. Neonates with a history of PDA ligation had a significantly higher risk of HL [OR = 4.92, 95% CI (2.43, 9.95), *I*^2^ = 0.00%, *P* = 0.00] (Supplementary Figure S10).

### 3.4 Other relevant risk factors

Craniofacial anomalies/congenital head and neck deformities (six studies) were significantly linked to HL [OR = 6.55, 95% CI (4.91, 8.73), *I*^2^ = 46.3%, *P* = 0.00] (Supplementary Figure S2).

Family history of HL (four studies) was identified as a key risk factor for HL [OR = 7.35, 95% CI (3.29, 16.45), *I*^2^ = 0.00%, *P* = 0.00] (Supplementary Figure S3).

Hyperbilirubinemia (eight studies) was significantly related to HL [OR = 3.11, 95% CI (2.24, 4.31), *I*^2^ = 57.3%, *P* = 0.00] (Supplementary Figure S4).

Intracranial hemorrhage (two studies) was significantly linked to HL [OR = 2.57, 95% CI (1.36, 4.84), *I*^2^ = 0.00%, *P* < 0.01] (Supplementary Figure S5).

Regarding duration of oxygen therapy (three studies), prolonged oxygen therapy increased HL risk [OR = 2.00, 95% CI (1.30, 3.09), *I*^2^ = 83.8%, *P* < 0.01] (Supplementary Figure S9).

Meningitis (two studies) was significantly linked to HL [OR = 2.06, 95% CI (1.21, 3.51), *I*^2^ = 0.00%, *P* < 0.01] (Supplementary Figure S12).

TORCH infection (two studies) was significantly correlated with HL [OR = 5.27, 95% CI (2.00, 13.93), *I*^2^ = 0.00%, *P* < 0.01] (Supplementary Figure S14).

Mechanical ventilation (seven studies) was linked to HL [OR = 0.75, 95% CI (0.53, 0.98), *I*^2^ = 60.1%, *P* = 0.00) (Supplementary Figure S8).

Postnatal hypoxia/severe birth asphyxia (two studies) was no significant linked to HL (OR = 0.81, 95%CI [0.42, 1.20], *I*^2^ = 68.7%, *P* = 0.07). (Figure S18).

### 3.5 Risk factors with No significant association

Low Apgar score (four studies) has no significant association with HL [OR = 0.96, 95% CI (0.81, 1.13), *I*^2^ = 70.1%, *P* = 0.62] (Supplementary Figure S7).

Prematurity/shorter gestational age (4 studies) is not significantly associated with HL [OR = −1.10, 95% CI (−0.31, 0.11), *I*^2^ = 71.9%, *P* = 0.34] (Supplementary Figure S11).

Sex (three studies) is not significantly associated with HL [OR = 0.99, 95% CI (0.77, 1.28), *I*^2^ = 0.0%, *P* = 0.93] (Supplementary Figure S21).

Sepsis (two studies) has no significant association with HL (OR = 1.03, 95%CI [0.73, 1.45], *I*^2^ = 0.00%, *P* = 0.85) (Figure S22).

Supplementary Figures S23–S44 show the overall and individual results of the risk of bias assessment. No publication bias was noted, so no further elimination or revision was conducted for these studies.

## 4 Discussion

The incidence of HL among neonates admitted to the NICU (1%–10%) is substantially higher than that observed in healthy neonates (approximately 0.1%) (1, 2). As the first comprehensive meta-analysis to systematically evaluate risk factors for HL in NICU neonates, this study provides robust evidence to inform clinical risk stratification and surveillance strategies. Our findings reinforce that genetic susceptibility and environmental injury constitute the two principal dimensions underlying HL in NICU neonates. Genetic risk is exemplified by a family history of HL and pathogenic variants like GJB2 mutations, with previous reports indicating that up to 56% of nonsyndromic HL cases have a positive family history (33). Environmental factors identified in this meta-analysis include prolonged mechanical ventilation and hyperoxia exposure, which may damage cochlear hair cells through hypoxia, oxidative stress, and excessive noise exposure (12, 34). In addition, hyperbilirubinemia (total serum bilirubin >10 mg/dL) exerts neurotoxic effects on retrocochlear auditory pathways, significantly increasing HL risk (35), supporting enhanced auditory monitoring in these neonates.

Subgroup analyses based on birth weight showed no significant association between low birth weight and hearing loss in NICU neonates, regardless of whether the birth weight was below 1,500 g or 1,500 g and above. This pattern may be related to the heterogeneity and limited number of included studies, as well as the potential confounding effects of concurrent perinatal adversities such as prematurity, hyperbilirubinemia, ototoxic drug exposure, and mechanical ventilation. Although very low birth weight infants are usually preterm, with immature cochlear development and increased vulnerability to hypoxia and oxidative stress, the pooled results did not reach statistical significance in the present meta-analysis (36, 37). Similarly, infants with a birth weight of 1,500 g or more also showed no statistically significant increase in hearing loss risk.

With respect to ototoxic medications, loop diuretics are widely used in the NICU for fluid management, yet their potential contribution to HL warrants careful consideration. Pooled analysis of studies by Eras, Hoog, and Robertson indicated that loop diuretic exposure may increase the risk of HL in premature NICU neonates. Experimental and clinical data suggest that intravenous administration at concentrations exceeding 50 μg/mL can alter endolymph composition and impair cochlear function (38, 39). Similarly, our meta-analysis confirmed a statistically significant association between aminoglycoside exposure and HL, reflecting their well-established ototoxic potential. Aminoglycosides induce cochlear injury primarily through disruption of cell membranes and interference with cellular metabolism (40). Ototoxicity varies by agent, with amikacin and netilmicin exhibiting relatively lower toxicity. Importantly, drug-induced HL is strongly modulated by genetic susceptibility, most notably the mitochondrial 12S rRNA m.1555A>G mutation, and by physiological vulnerability associated with prematurity and systemic inflammation, which can markedly amplify ototoxic effects (41). In adult studies, Vancomycin's HL risk shows a concentration dependence feature (42). When the serum concentration exceeds 40 mg/L, it may cause temporary SNHL, while a concentration of ≥80 mg/L may lead to permanent damage (43). Given the prolonged drug clearance and heightened vulnerability of extremely low birth weight infants, particularly those <1,000 g, subgroup analyses stratified by birth weight are likely essential for accurately evaluating vancomycin-associated HL risk in neonates. However, current evidence remains insufficient, highlighting a critical gap in the literature.

Beyond pharmacological factors, surgical ligation of PDA emerged as an important risk factor for HL. This association may reflect the combined effects of anesthetic neurotoxicity, intraoperative cerebral hemodynamic fluctuations, and underlying congenital vulnerabilities. The impact appears especially pronounced in extremely premature infants, consistent with previous reports (19).

This study has several notable strengths and innovations. First, it systematically validates the applicability of the JCIH risk indicators within the NICU population, clearly demonstrating the clinical relevance of core factors like craniofacial anomalies and family history of HL. Second, it expands the existing risk factor framework by identifying specific ototoxic agents (loop diuretics, aminoglycosides, and vancomycin) and NICU-specific interventions (e.g., PDA surgical ligation) not explicitly addressed in current JCIH guidelines, thereby filling an important gap in neonatal HL risk assessment.

Nevertheless, several limitations should be acknowledged. The limited number of eligible studies precluded subgroup analyses distinguishing sensorineural from conductive HL. Additionally, the small sample sizes in studies examining vancomycin exposure and PDA ligation possibly cause bias. Due to insufficient data, the association between small-for-gestational-age status and HL could not be evaluated, nor could the potential synergistic ototoxic effects of combined aminoglycoside and diuretic therapy be assessed. Future research should prioritize large-scale, multicenter prospective studies to elucidate the synergistic pathogenic interactions among genetic susceptibility, ototoxic medications, and hypoxic injury. Such studies are essential to define safe exposure thresholds for interventions like vancomycin administration and PDA surgery and to establish a more comprehensive, evidence-based risk assessment framework for HL in NICU neonates.

## Data Availability

The original contributions presented in the study are included in the article/Supplementary Material, further inquiries can be directed to the corresponding author.
