# Correction: Birth weight, ototoxic medication, and surgical history predict individual hearing loss risks: a systematic review and meta-analysis

**DOI:** 10.3389/fped.2026.1947631

**Published:** 2026-08-12

**Authors:** Hanwen Luo, Jianghua He, Dapeng Chen, Xiaoming Xu, Jing Zhao, Xiaoyan Yang, Jing Shi

**Affiliations:** 1 Department of Pediatrics, West China Second University Hospital, Chengdu, Sichuan, China; 2 Key Laboratory of Birth Defects and Related Diseases of Women and Children, Ministry of Education, Sichuan University, Chengdu, Sichuan, China

**Keywords:** HL, newborns, NICU, ototoxic medication, surgical history

There were errors in multiple sections of the text, the corrected text appears below.

A correction has been made to the Abstract.

“Meta-analysis indicated that exposure to ototoxic drugs (aminoglycosides, loop diuretics), history of the surgical ligation of patent ductus arteriosus (PDA), craniofacial anomalies, family history of HL, and TORCH infections were significantly associated with HL in NICU neonates (all *P* ≤ 0.05). In contrast, low Apgar score, prematurity, low birth weight, duration of vancomycin exposure, sex, and sepsis were not significantly correlated.”

“Statistically significant risk factors for HL included craniofacial anomalies, family history of HL, TORCH infections, and surgical ligation of PDA, as well as hyperbilirubinemia, exposure to loop diuretics and other ototoxic drugs, and mechanical ventilation (all *P* ≤ 0.05).”

A correction has been made to the Results section.

“Very low birth weight (<1,500 g): Data from 4 studies indicated no significant risk of HL in this weight group (OR = 3.05, 95%CI [0.57, 16.38], *I*^2^ = 72.8%, *P* = 0.19).”

“Postnatal hypoxia/severe birth asphyxia (two studies) was no significant linked to HL (OR = 0.81, 95%CI [0.42, 1.20], *I*^2^ = 68.7%, *P* = 0.07). (Figure S18)”

“Sepsis (two studies) has no significant association with HL (OR = 1.03, 95%CI [0.73, 1.45], *I*^2^ = 0.00%, *P* = 0.85). (Figure S22)”

A correction has been made to the Discussion section.

“Subgroup analyses based on birth weight showed no significant association between low birth weight and hearing loss in NICU neonates, regardless of whether the birth weight was below 1,500 g or 1,500 g and above. This pattern may be related to the heterogeneity and limited number of included studies, as well as the potential confounding effects of concurrent perinatal adversities such as prematurity, hyperbilirubinemia, ototoxic drug exposure, and mechanical ventilation. Although very low birth weight infants are usually preterm, with immature cochlear development and increased vulnerability to hypoxia and oxidative stress, the pooled results did not reach statistical significance in the present meta-analysis (36, 37). Similarly, infants with a birth weight of 1,500 g or more also showed no statistically significant increase in hearing loss risk.”

Error in supplementary material.

Revised forest plots for Figure S17 and Figure S22 have been provided as supplementary files in this submission, replacing the original figures.

The original article has been updated.

