# Supplementary material for: Birth weight, ototoxic medication, and surgical history predict individual hearing loss risks: a systematic review and meta-analysis
Source: Front Pediatr. 2026 Feb 6;14:1729458. doi: 10.3389/fped.2026.1729458 (PMC12920535; doi:10.3389/fped.2026.1729458)
Supplement: Supplementary file 1 [file Supplementaryfile1.docx]

**Supplementary file 1** Search strategy

1. (Infant, Newborn [Title/Abstract]) OR (Infants, Newborn [Title/Abstract]) OR (Newborn Infant [Title/Abstract]) OR (Newborn Infants [Title/Abstract]) OR (Newborns [Title/Abstract]) OR (Newborn [Title/Abstract]) OR (Neonate [Title/Abstract]) OR (Neonates [Title/Abstract]) OR (neonatal [Title/Abstract]) OR ("Infant, Newborn" [Mesh])
2. (Loss, Hearing [Title/Abstract]) OR (Hypoacusis [Title/Abstract])) OR (hearing loss [Title/Abstract]) OR (Hearing Impairment [Title/Abstract]) OR (Deafness, Transitory [Title/Abstract]) OR (Deafnesses, Transitory [Title/Abstract]) OR (Transitory Deafness [Title/Abstract]) OR (Transitory Deafnesses [Title/Abstract]) OR (Transitory Hearing Loss [Title/Abstract]) OR (Hearing Loss, Transitory [Title/Abstract]) OR (Loss, Transitory Hearing [Title/Abstract]) OR (Transitory Hearing Losses [Title/Abstract]) OR ("Hearing Loss" [Mesh])
3. ("Risk Factors" [Mesh]) OR (Factor, Risk [Title/Abstract]) OR (Risk Factor [Title/Abstract]) OR (Social Risk Factors [Title/Abstract]) OR (Factor, Social Risk [Title/Abstract]) OR (Factors, Social Risk [Title/Abstract]) OR (Risk Factor, Social [Title/Abstract]) OR (Risk Factors, Social [Title/Abstract]) OR (Social Risk Factor [Title/Abstract]) OR (Health Correlates [Title/Abstract]) OR (Correlates, Health [Title/Abstract]) OR (Population at Risk [Title/Abstract]) OR (Populations at Risk [Title/Abstract]) OR (Risk Scores [Title/Abstract]) OR (Risk Score [Title/Abstract]) OR (Score, Risk [Title/Abstract]) OR (Risk Factor Scores [Title/Abstract]) OR (Risk Factor Score [Title/Abstract]) OR (Score, Risk Factor [Title/Abstract]) OR (Risk factors [Title/Abstract])
4. ("Ventilation" [Mesh]) OR (Ventilation [Title/Abstract])
5. ("Ototoxicity" [Mesh]) OR (Drug-Induced Ototoxicity [Title/Abstract]) OR (Drug Induced Ototoxicity [Title/Abstract]) OR (Ototoxicity, Drug-Induced [Title/Abstract]) OR (Drug-Related Otological Toxicity [Title/Abstract]) OR (Drug Related Otological Toxicity [Title/Abstract]) OR (Drug-Related Otological Toxicities [Title/Abstract]) OR (Otological Toxicity, Drug-Related [Title/Abstract]) OR (Drug-Induced Otological Toxicity [Title/Abstract]) OR (Drug Induced Otological Toxicity [Title/Abstract])
6. (neurodevelopmental outcome [Title/Abstract])
7. 1# AND 2# AND 3#
8. 1# AND 2# AND 4#
9. 1# AND 2# AND 5#
10. 1# AND 2# AND 6#

**Table S1** Quality assessment results

| **Result** | **included studies** | **OR** | **95%CI** | **I^2** | **P** |
| --- | --- | --- | --- | --- | --- |
| Ototoxic medication | Richard （1992） | OR=2.00 | 95%CI[1.52,2.63] | I^2=65.2% | P=0.00 |
|  | Maharani（2015） |  |  |  |  |
|  | Wang（2017） |  |  |  |  |
|  | Khairy（2018） |  |  |  |  |
|  | ABDULLAH（2020） |  |  |  |  |
| Craniofacial anomalies/ Congenital head and neck deformity | Hajare（2021） | OR=6.55 | 95%CI[4.91,8.73] | I^2=46.3% | P=0.00 |
|  | Richard （1992） |  |  |  |  |
|  | Dommelen（2010） |  |  |  |  |
|  | ABDULLAH（2020） |  |  |  |  |
|  | Wang（2017） |  |  |  |  |
|  | Lima（2006） |  |  |  |  |
|  | Nair2021 |  |  |  |  |
| Family history | Richard （1992） | OR=7.35 | 95%CI[3.29,16.45] | I^2=0.00% | P=0.00 |
|  | Lima（2006） |  |  |  |  |
|  | Bhat(2018) |  |  |  |  |
|  | Hajare（2021） |  |  |  |  |
| Hyperbilirubinemia | Gupta(1991) | OR=3.11 | 95%CI[2.24,4.31] | I^2=57.3% | P=0.00 |
|  | Richard （1992） |  |  |  |  |
|  | Lima（2006） |  |  |  |  |
|  | Alaee(2015) |  |  |  |  |
|  | Maharani(2015) |  |  |  |  |
|  | Bhat(2018) |  |  |  |  |
|  | ABDULLAH（2020） |  |  |  |  |
|  | Nair（2021） |  |  |  |  |
|  | Hajare（2021） |  |  |  |  |
| Intracranial hemorrhage | Wang（2017） | OR=2.57 | 95%CI[1.36,4.84] | I^2=0.00% | P<0.01 |
|  | Hajare（2021） |  |  |  |  |
| Loop diuretics | HOOG(2003) | OR=3.26 | 95%CI[2.04,5.14] | I^2=50.0% | P=0.00 |
|  | Eras(2014) |  |  |  |  |
|  | Chant（2022） |  |  |  |  |
|  | ROBERTSON(2005) |  |  |  |  |
| low apgar score | Richard （1992） | OR=0.96 | 95%CI[0.81,1.13] | I^2=70.1% | P=0.62 |
|  | Dommelen（2010） |  |  |  |  |
|  | Bhat(2018) |  |  |  |  |
|  | ABDULLAH（2020） |  |  |  |  |
|  | Chant（2022） |  |  |  |  |
| Mechanical ventilation | Lima（2006） | OR=0.75 | 95%CI[0.53,0.98] | I^2=60.1% | P=0.00 |
|  | Hille(2007) |  |  |  |  |
|  | Dommelen（2010） |  |  |  |  |
|  | Eras(2014) |  |  |  |  |
|  | Khairy（2018） |  |  |  |  |
|  | ABDULLAH（2020） |  |  |  |  |
|  | Hajare（2021） |  |  |  |  |
|  | Nair（2021） |  |  |  |  |
| Oxygen duration | LESLIE(1995) | OR=2.00 | 95%CI[1.30,3.09] | I^2=83.8% | P<0.01 |
|  | Dommelen（2010） |  |  |  |  |
|  | Wang（2017） |  |  |  |  |
| PDA surgical ligation | Eras(2014) | OR=4.92 | 95%CI[2.43,9.95] | I^2=0.00% | P=0.00 |
|  | Wang（2017） |  |  |  |  |
| Prematurity /Shorter gestational length | Gupta(1991) | OR=0.96 | 95%CI[0.80,1.15] | I^2=67.4% | P=0.64 |
|  | Arslan（2013） |  |  |  |  |
|  | Alaee(2015) |  |  |  |  |
|  | Chant（2022） |  |  |  |  |
|  | Wang（2017） |  |  |  |  |
| Meningitis | Richard （1992） | OR=2.06 | 95%CI[1.21,3.51] | I^2=0.00% | P<0.01 |
|  | Dommelen（2010） |  |  |  |  |
|  | Nair（2021） |  |  |  |  |
|  | Maharani(2015) |  |  |  |  |
| Aminoglycoside | HOOG(2003) | OR=1.95 | 95%CI[1.35,2.82] | I^2=74.8% | P<0.01 |
|  | Cooper(2011) |  |  |  |  |
|  | Alaee(2015) |  |  |  |  |
|  | Nair（2021） |  |  |  |  |
|  | Alaee(2015) |  |  |  |  |
| Torch infection | Dommelen（2010） | OR=5.27 | 95%CI[2.00,13.93] | I^2=0.00% | P<0.01 |
|  | Hajare（2021） |  |  |  |  |
| low birth weight（Total） | Gupta(1991) | OR=1.31 | 95%CI[0.99,1.74] | I^2=65.6% | P=0.06 |
|  | Lima（2006） |  |  |  |  |
|  | Dommelen（2010） |  |  |  |  |
|  | Alaee(2015) |  |  |  |  |
|  | ABDULLAH（2020） |  |  |  |  |
|  | Hajare（2021） |  |  |  |  |
|  | Wang（2017） |  |  |  |  |
|  | Chant（2022） |  |  |  |  |
| low birth weight（1500-2500g） | Dommelen（2010） | OR=1.29 | 95%CI[0.79,2.13] | I^2=15.0% | P=0.31 |
|  | Hajare（2021） |  |  |  |  |
| low birth weight（<1500g） | Gupta(1991) | OR=0.94 | 95%CI[0.22,1.66] | I^2=72.8% | P=0.01 |
|  | Lima（2006） |  |  |  |  |
|  | Dommelen（2010） |  |  |  |  |
|  | Alaee(2015) |  |  |  |  |
|  | Wang（2017） |  |  |  |  |
|  | Chant（2022） |  |  |  |  |
| Postnatal hypoxia/severe birth asphyxia. | Lima（2006） | OR=0.81 | 95%CI[0.42,1.20] | I^2=68.7% | P=0.00 |
|  | Hille(2007) |  |  |  |  |
| Vancomycin | HOOG(2003) | OR=1.65 | 95%CI[0.97,2.82] | I^2=84.9% | P=0.06 |
|  | Nair（2021） |  |  |  |  |
|  | Alaee(2015) |  |  |  |  |
| Vancomycin duration | HOOG(2003) | OR=1.32 | 95%CI[0.89,1.96] | I^2=78.4% | P=0.17 |
|  | Alaee(2015) |  |  |  |  |
|  | Nair（2021） |  |  |  |  |
| Sex | Dommelen（2010） | OR=0.99 | 95%CI[0.77,1.28] | I^2=0.00% | P=0.93 |
|  | Chant（2022） |  |  |  |  |
|  | Hajare（2021） |  |  |  |  |
| Sepsis | Dommelen（2010） | OR=0.03 | 95%CI[-0.31,0.37] | I^2=0.00% | P=0.85 |
|  | Nair（2021） |  |  |  |  |


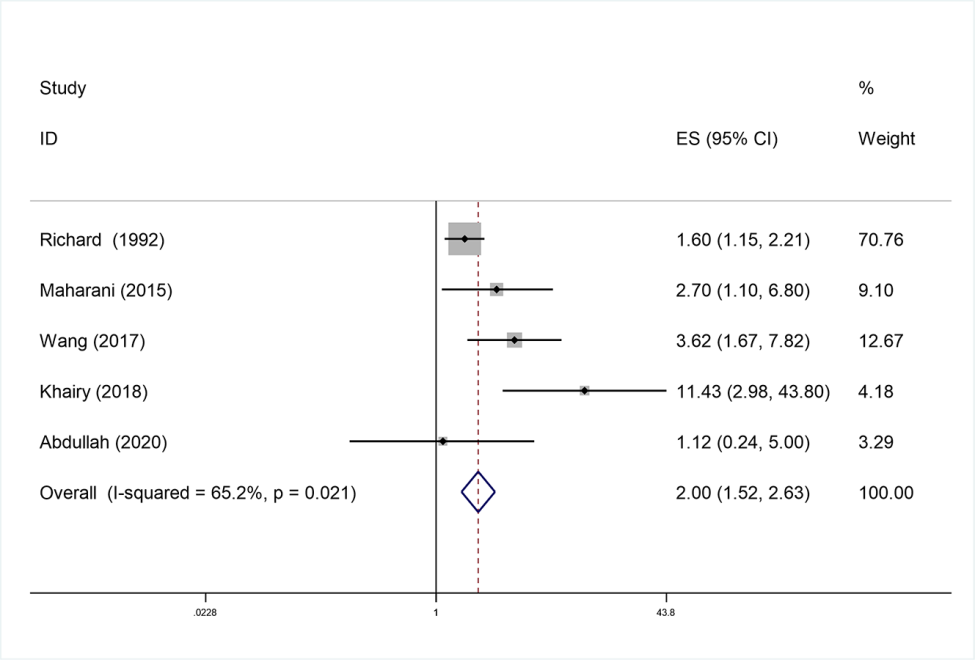


**Figure S1** Ototoxic medication


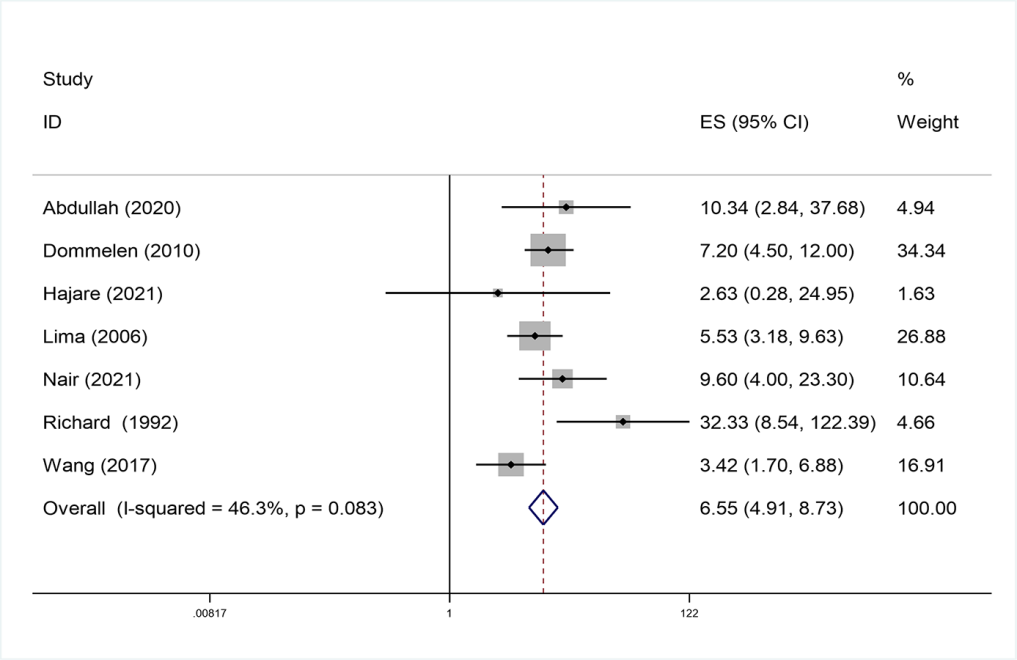


**Figure S2** Craniofacial anomalies


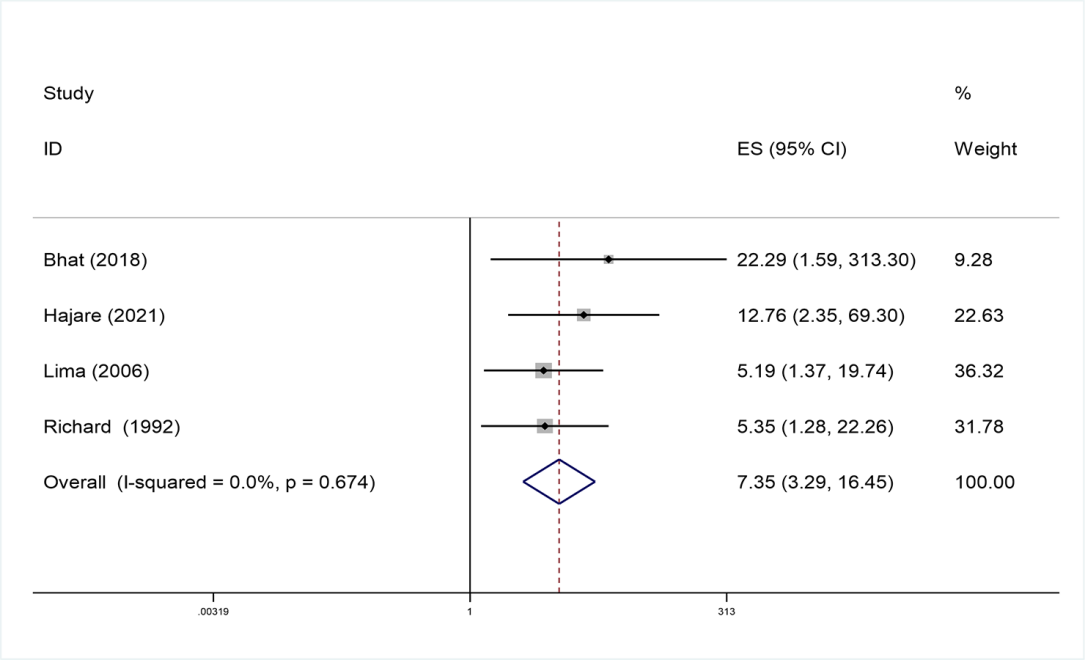


**Figure S3** Family history


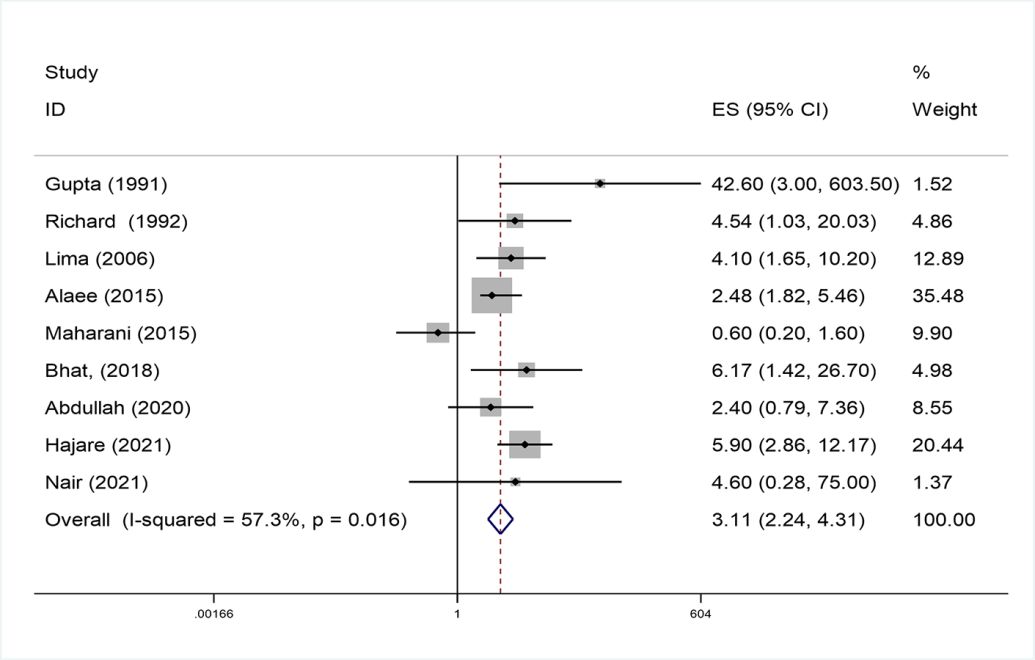


**Figure S4** Hyperbilirubinemia


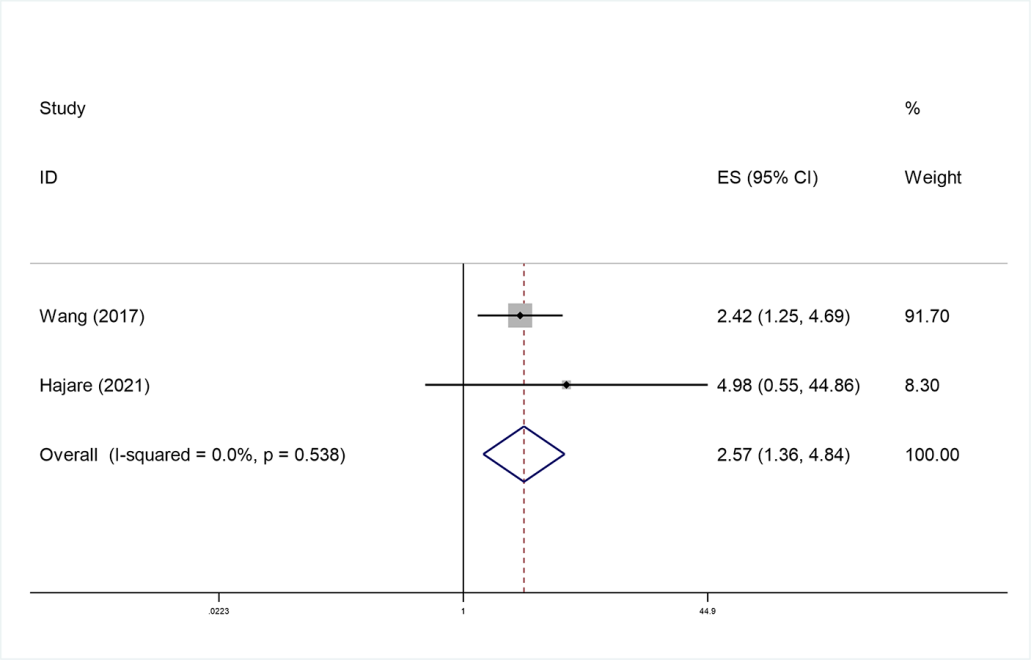


**Figure S5** Intracranial hemorrhage


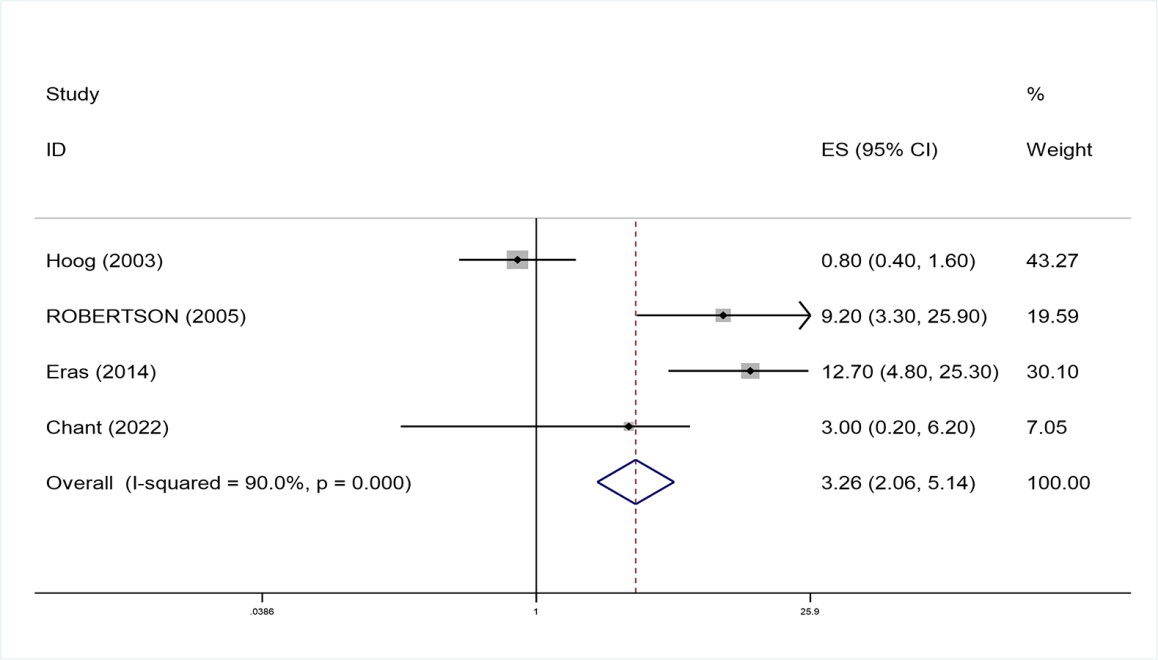


**Figure S6** Loop diuretics


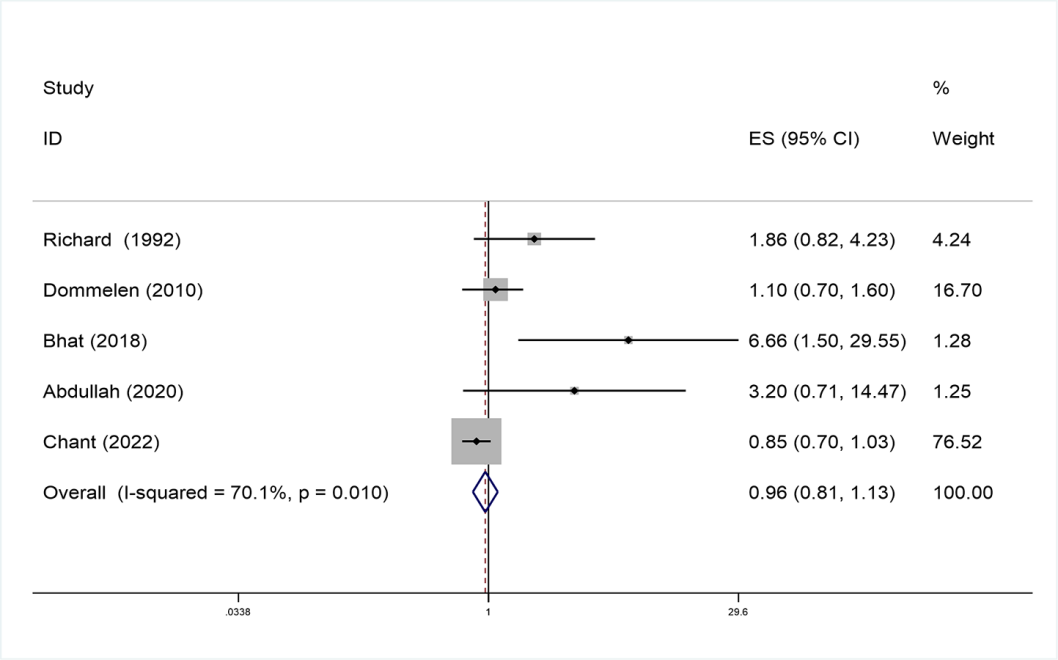


**Figure S7** Low Apgar scores


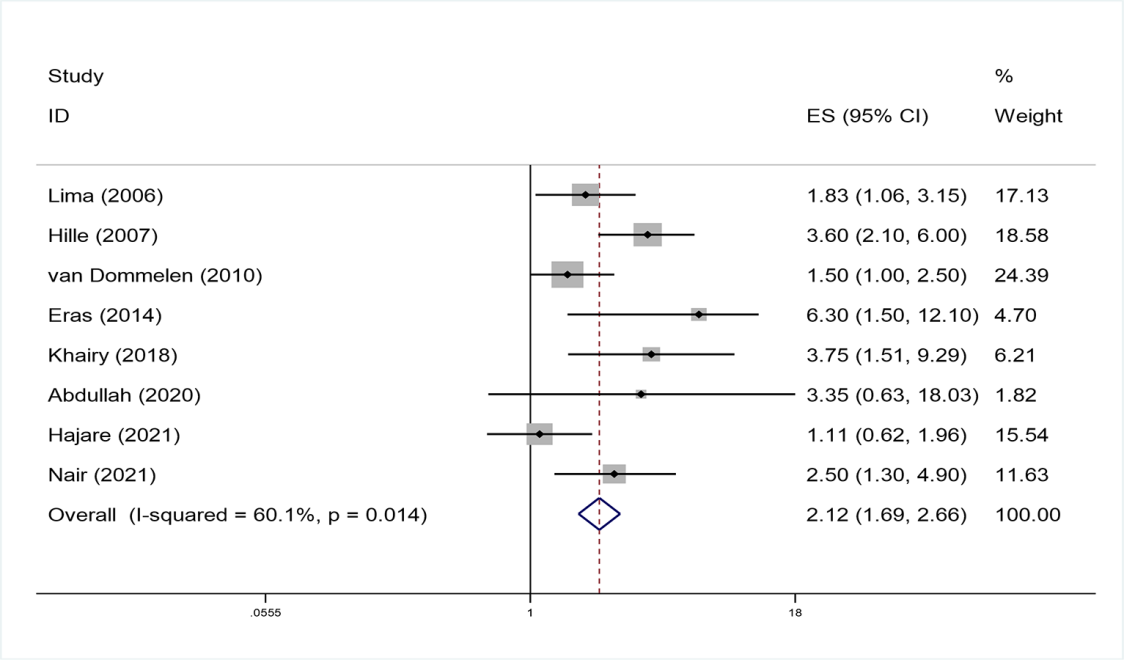


**Figure S8** Mechanical ventilation


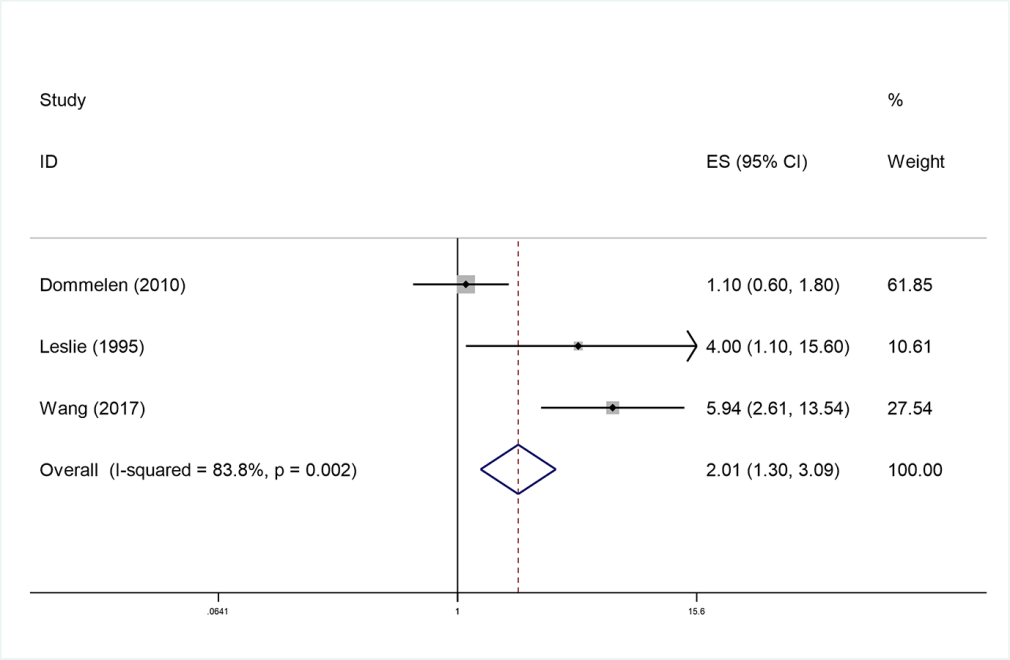


**Figure S9** Oxygen duration


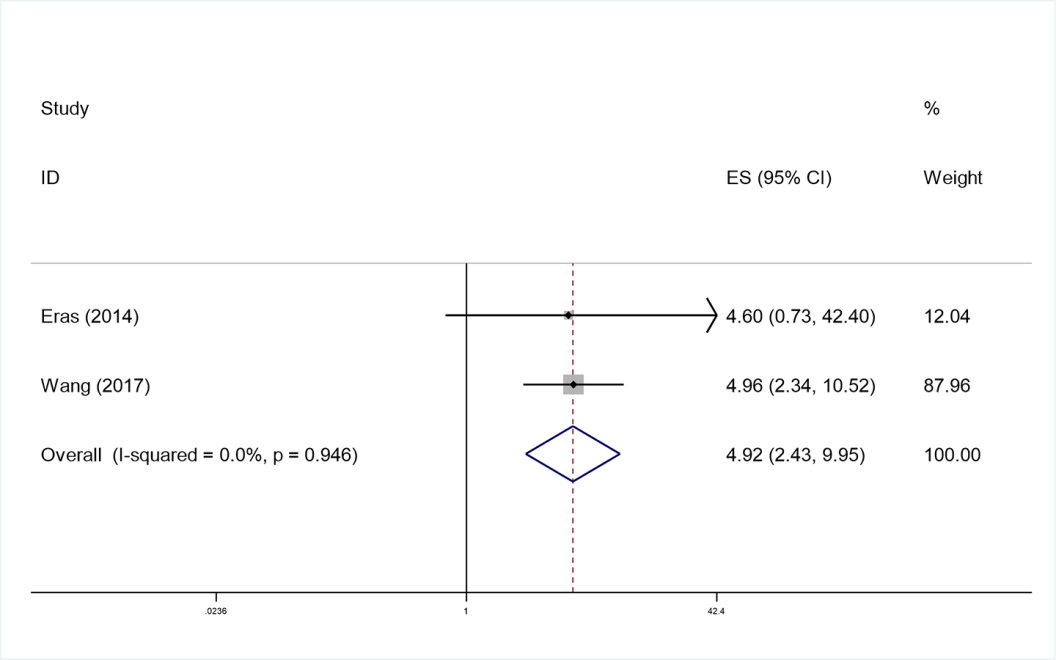


**Figure S10** PDA ligation


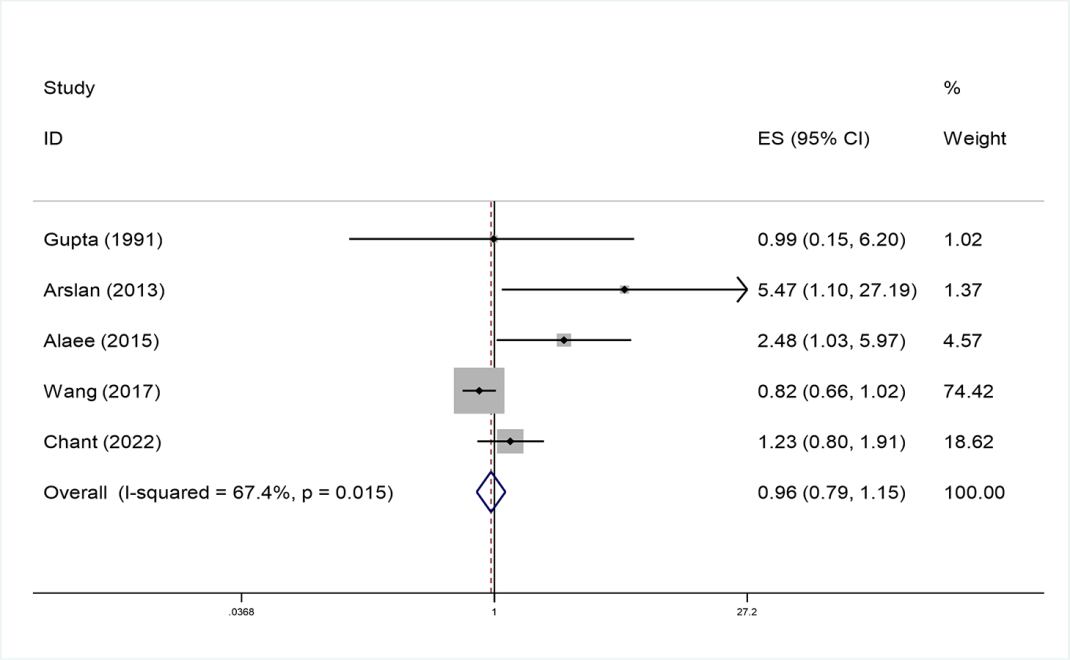


**Figure S11** Prematurity/Shorter gestational length


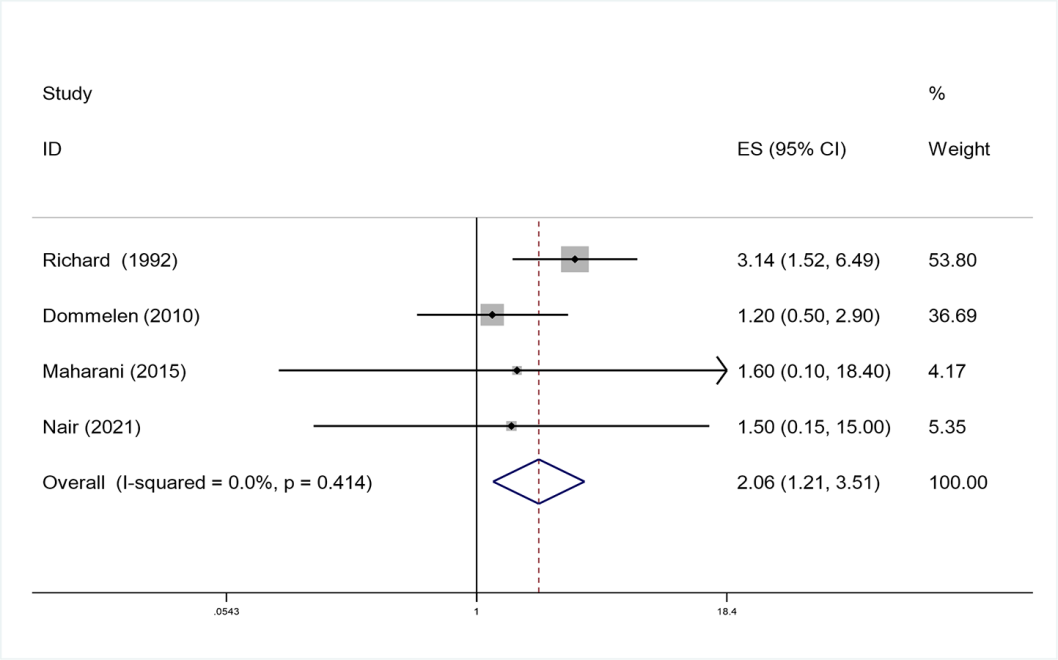


**Figure S12** Meningitis


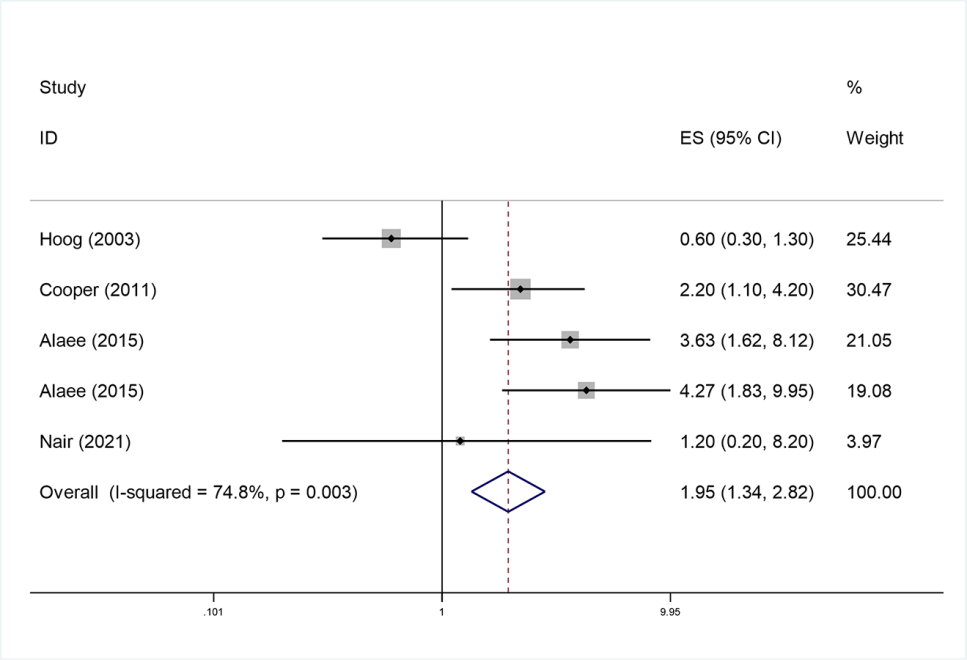


**Figure S13** aminoglycoside


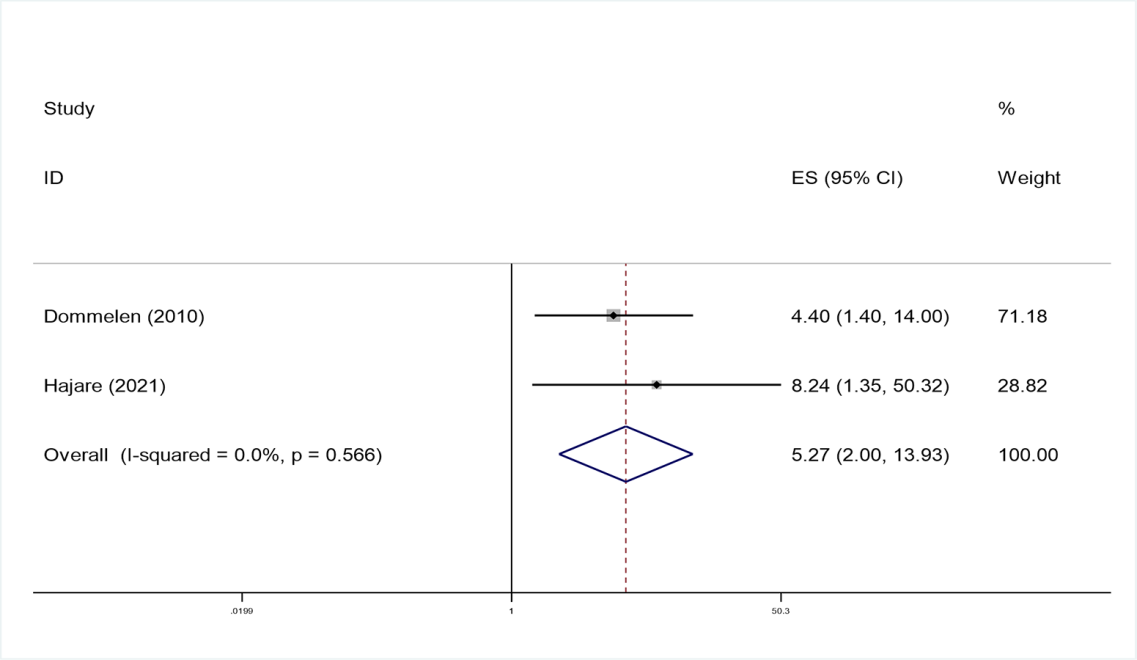


**Figure S14** TORCH infection


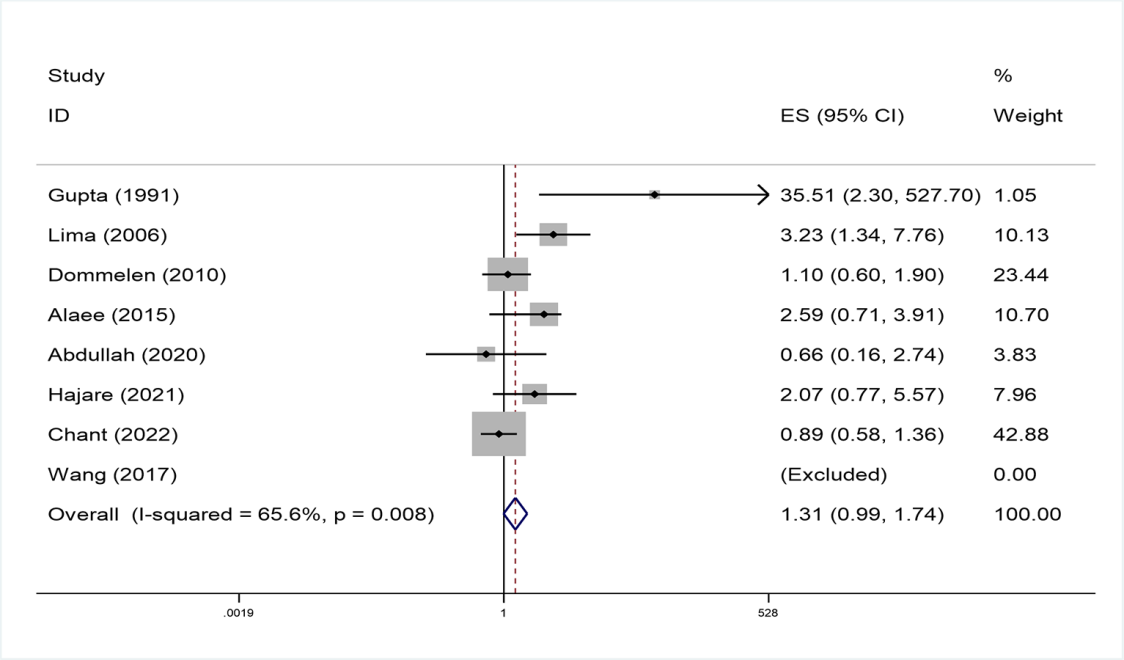


**Figure S15** low birth weight (Total)


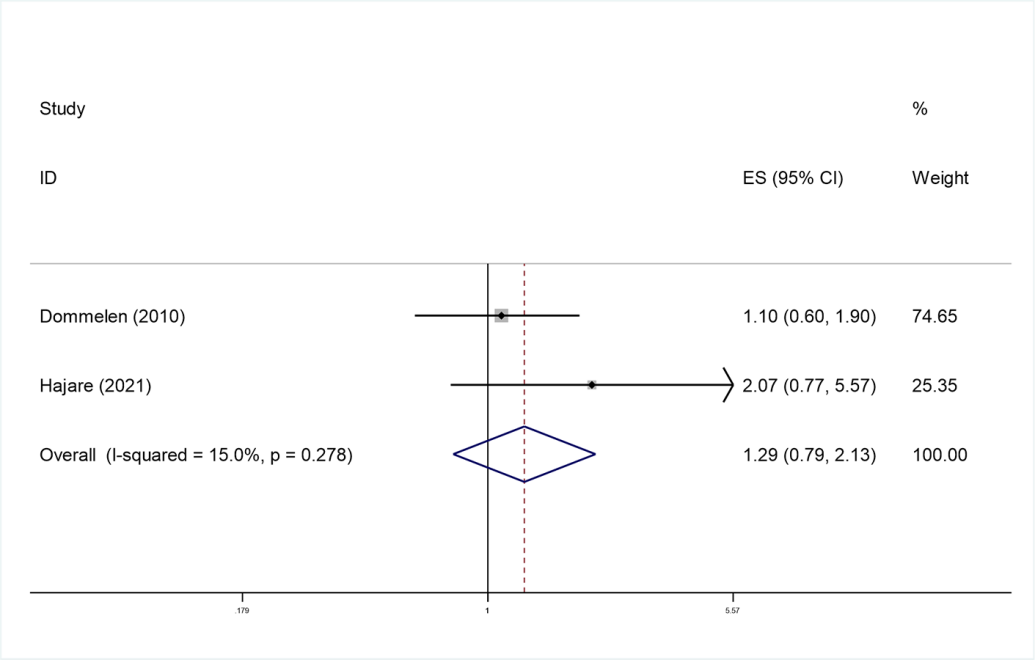


**Figure S16** low birth weight（from 1500 to 2500g）


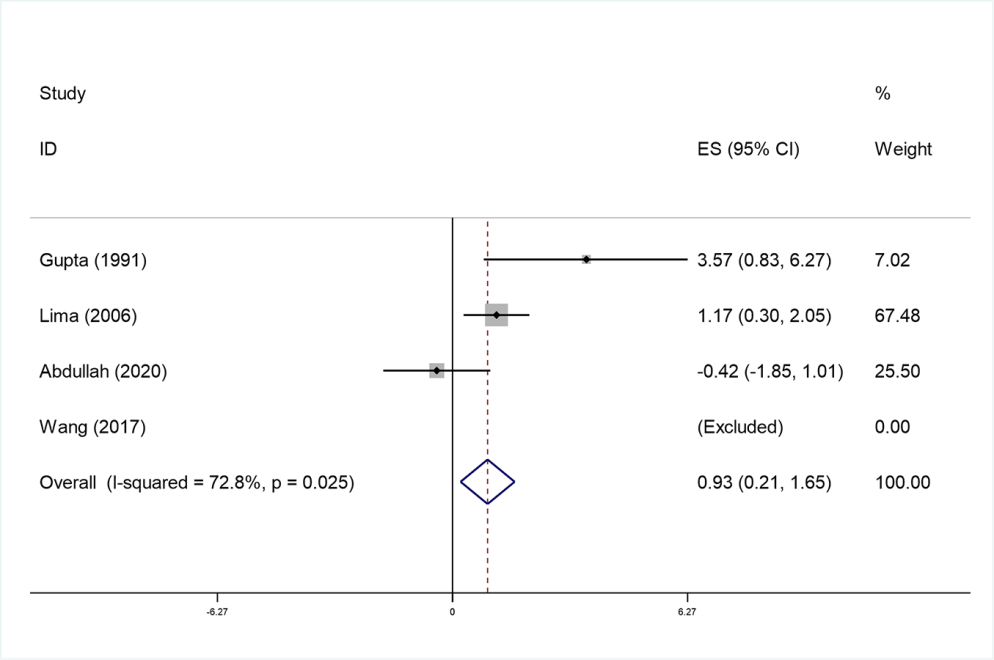


**Figure S17** low birth weight（<1500g）


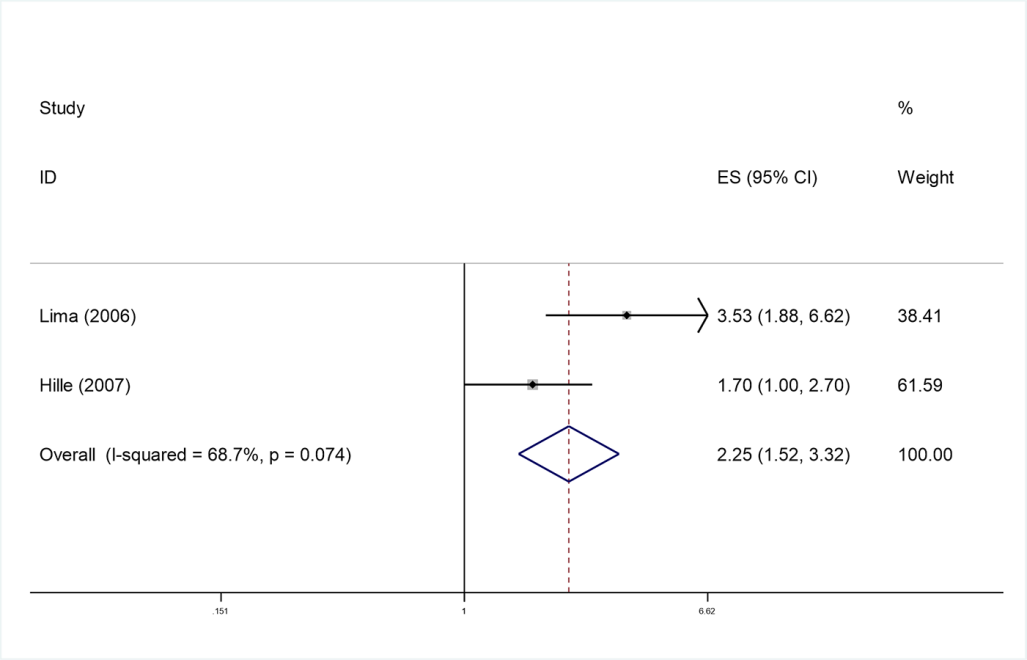


**Figure S18** Postnatal hypoxia


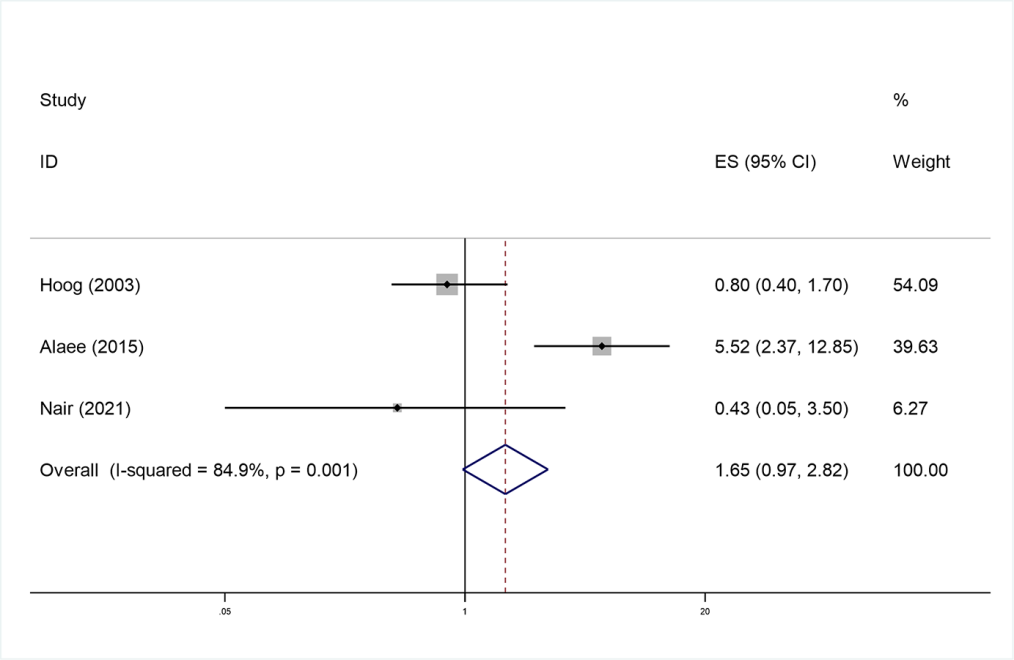


**Figure S19** Vancomycin


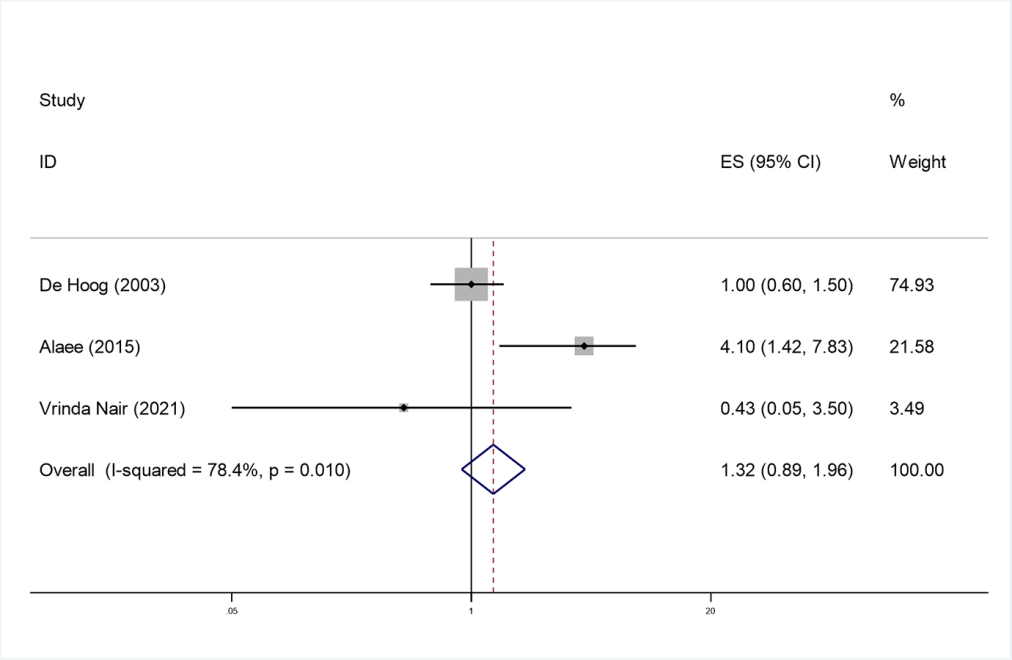


**Figure S20** Duration of Vancomycin


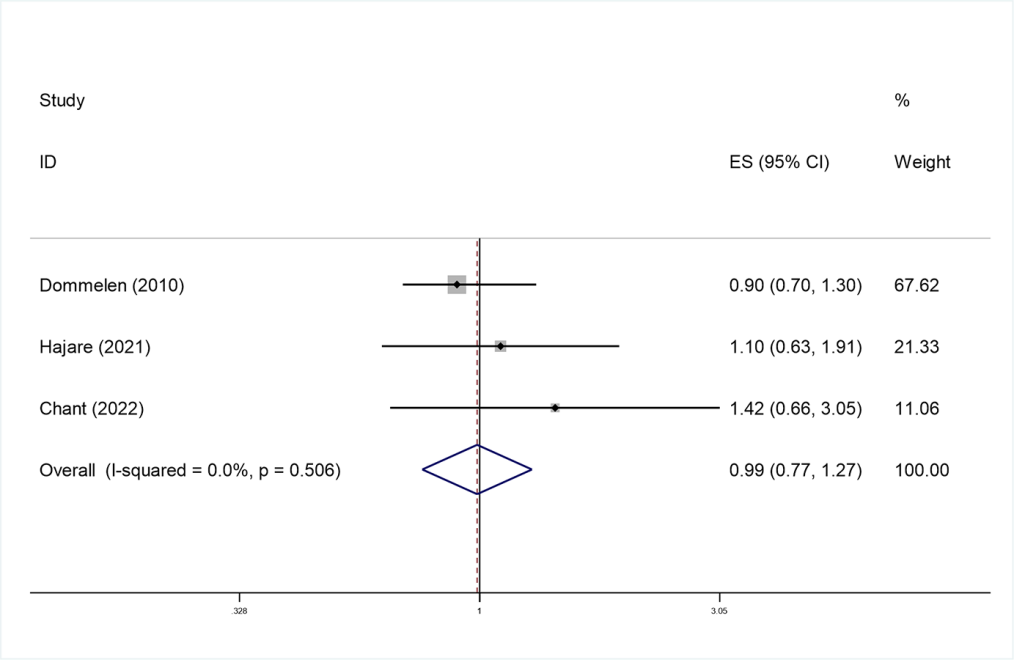


**Figure S21** Sex


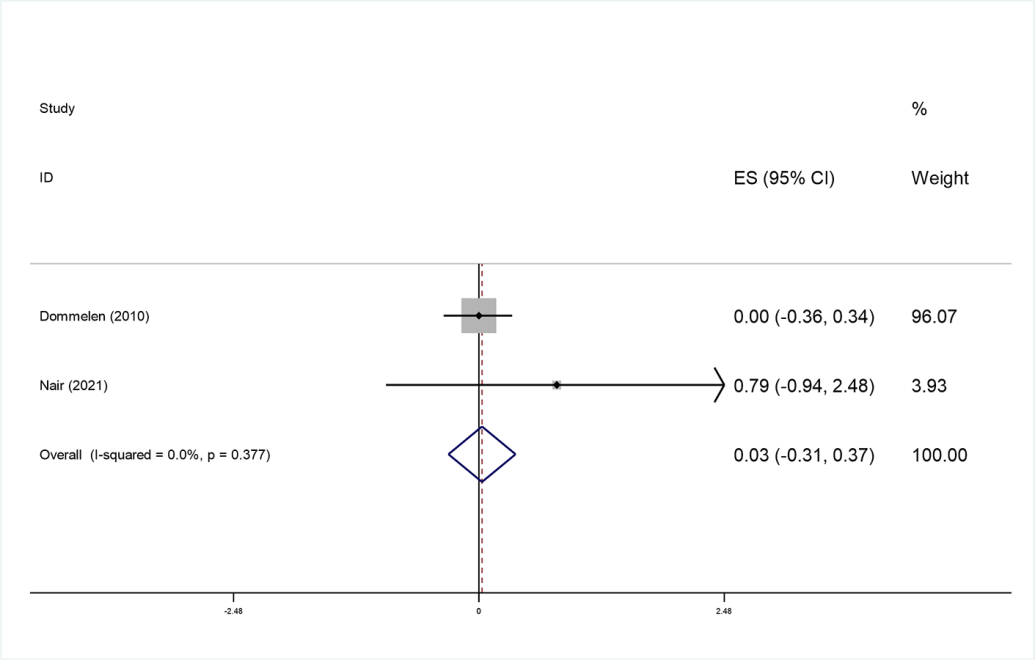


**Figure S22** Sepsis

**
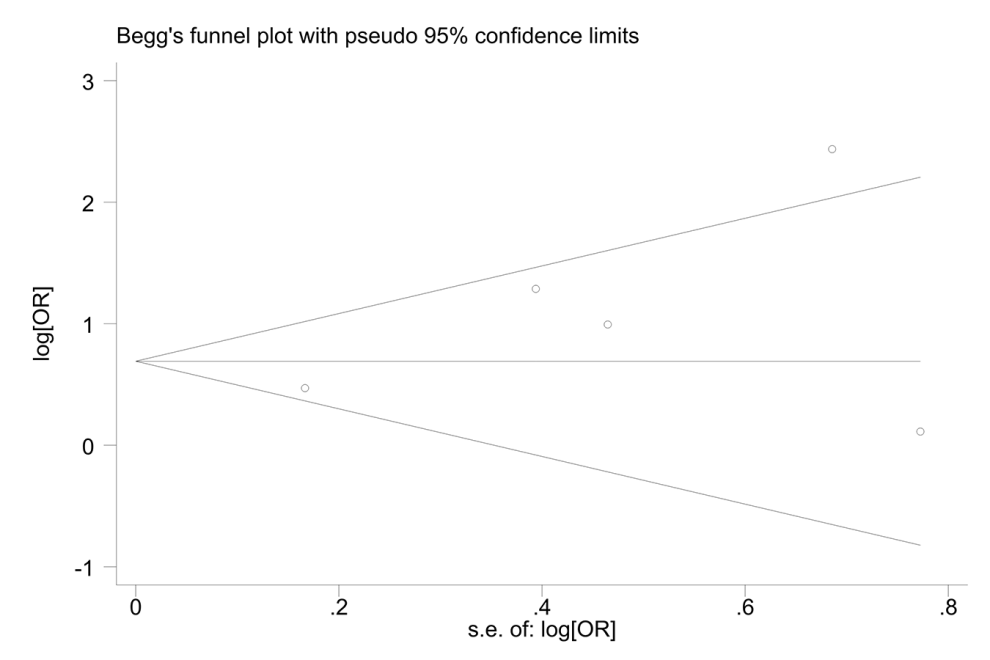
**

**Figure S23** Ototoxic medication

**
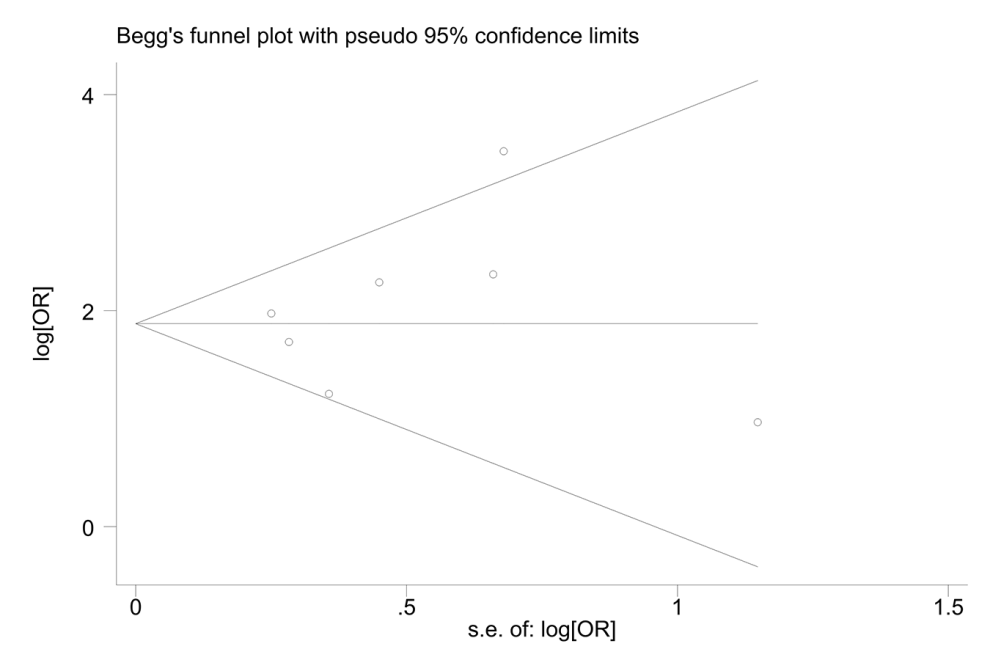
**

**Figure S24** Craniofacial anomalies

**
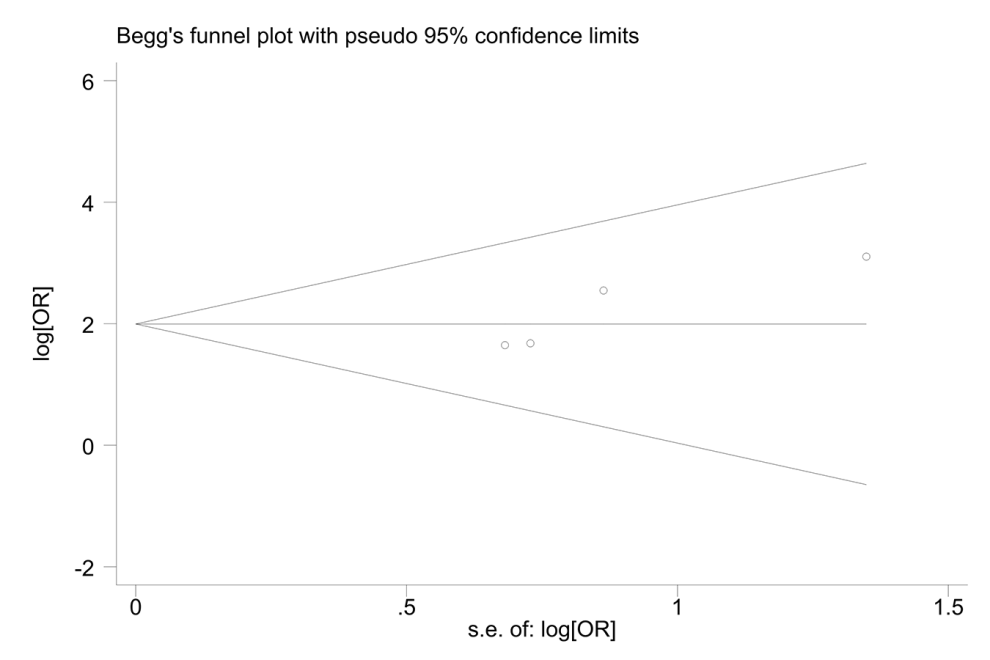
**

**Figure S25** Family history

**
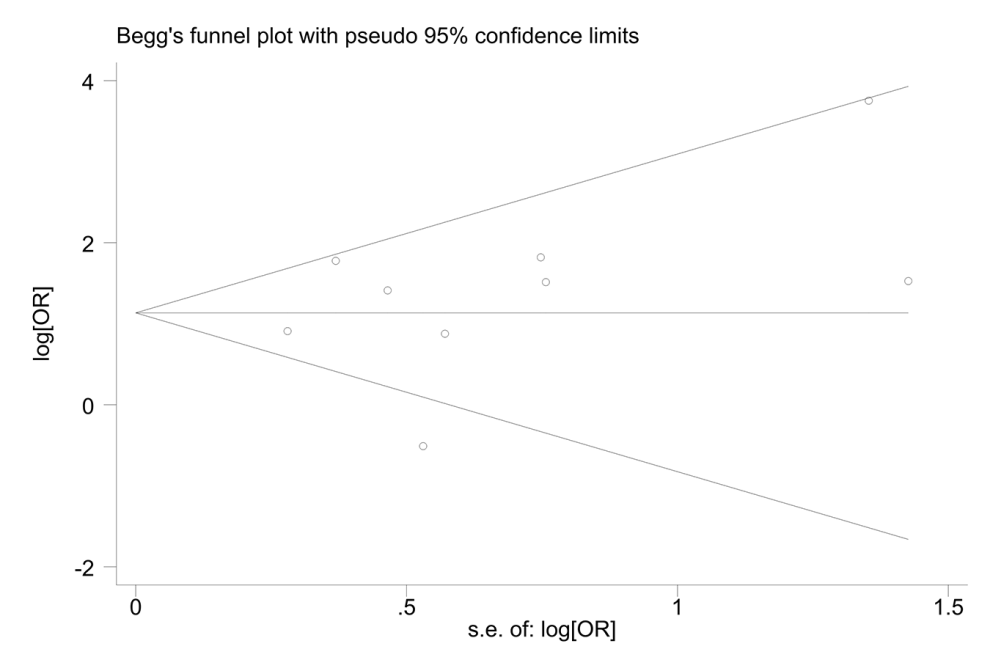
**

**Figure S26** Hyperbilirubinemia

**
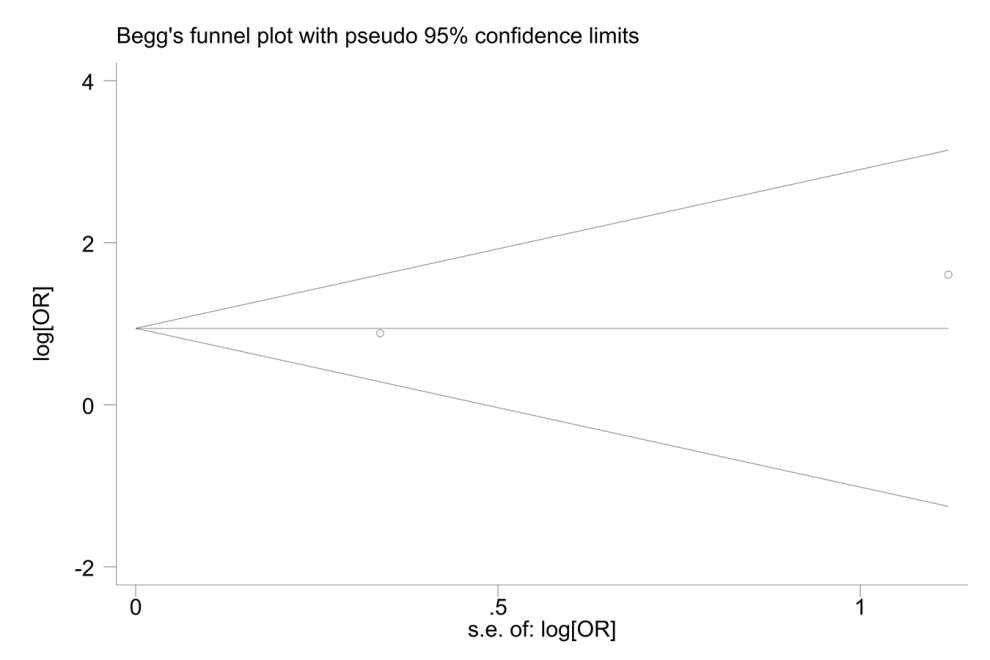
**

**Figure S27** Intracranial hemorrhage

**
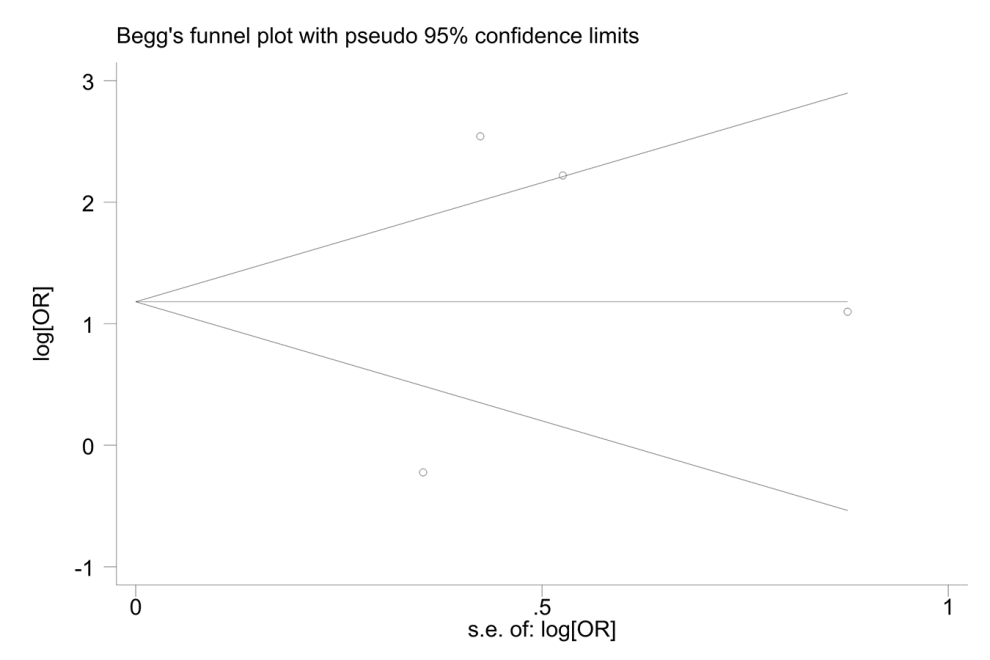
**

**Figure S28** Loop diuretics

**
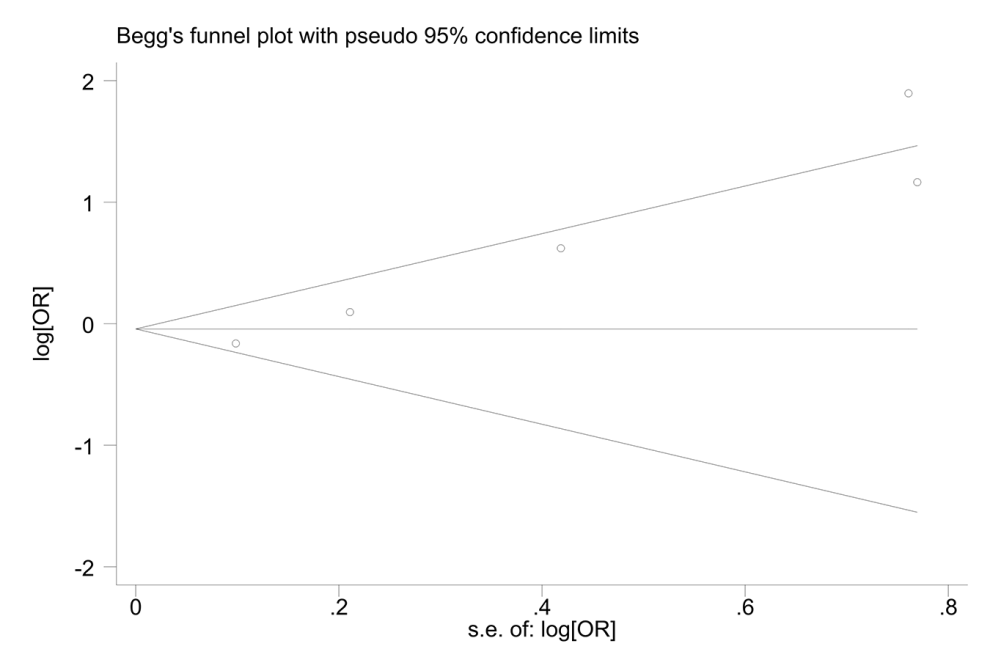
**

**Figure S29** Low Apgar scores

**
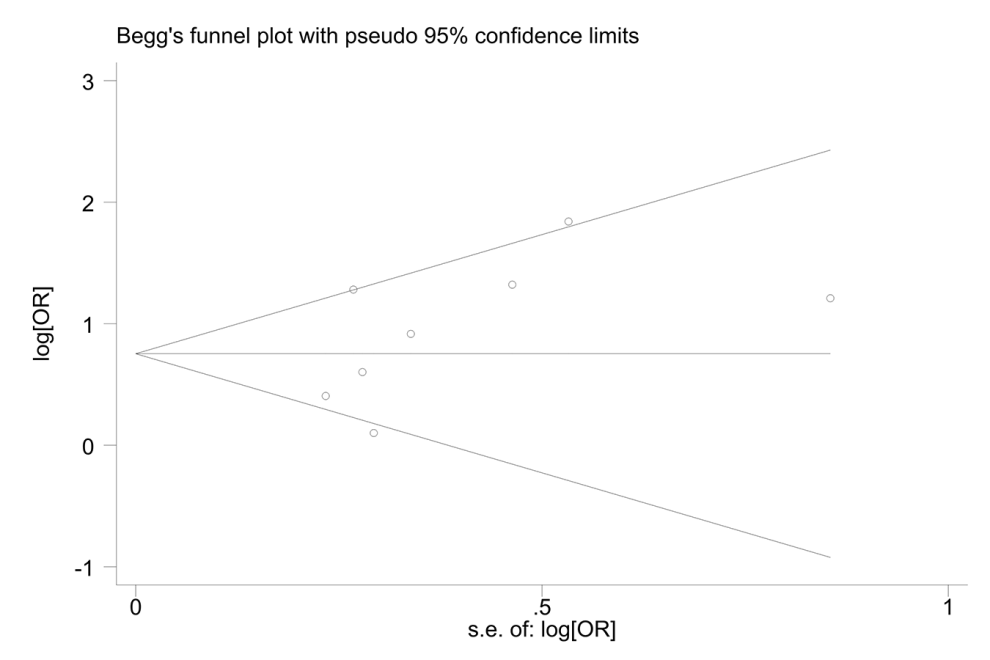
**

**Figure S30** Mechanical ventilation

**
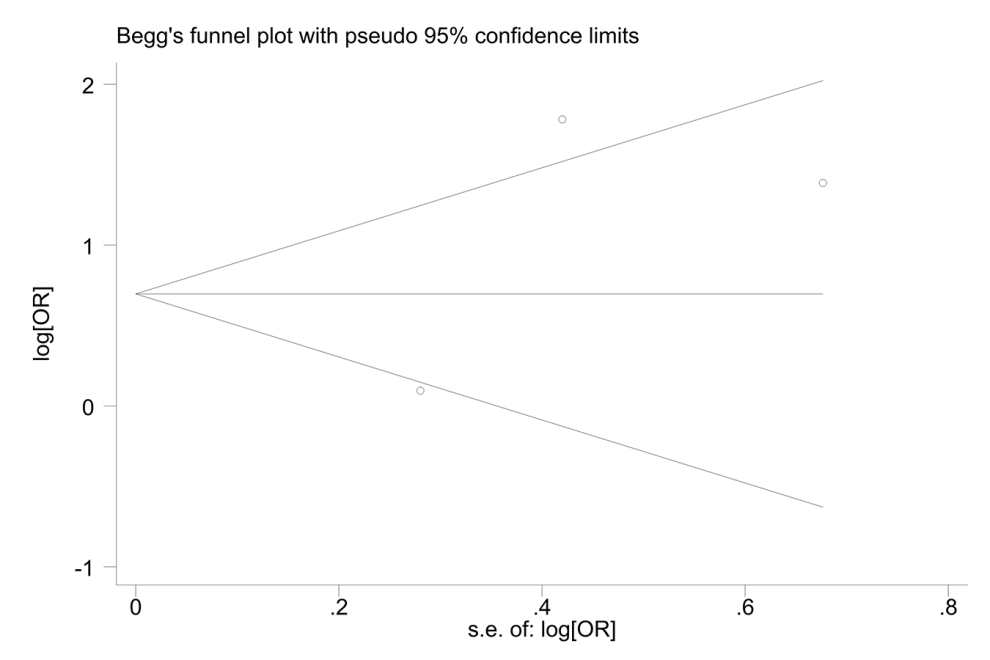
**

**Figure S31** Oxygen duration

**
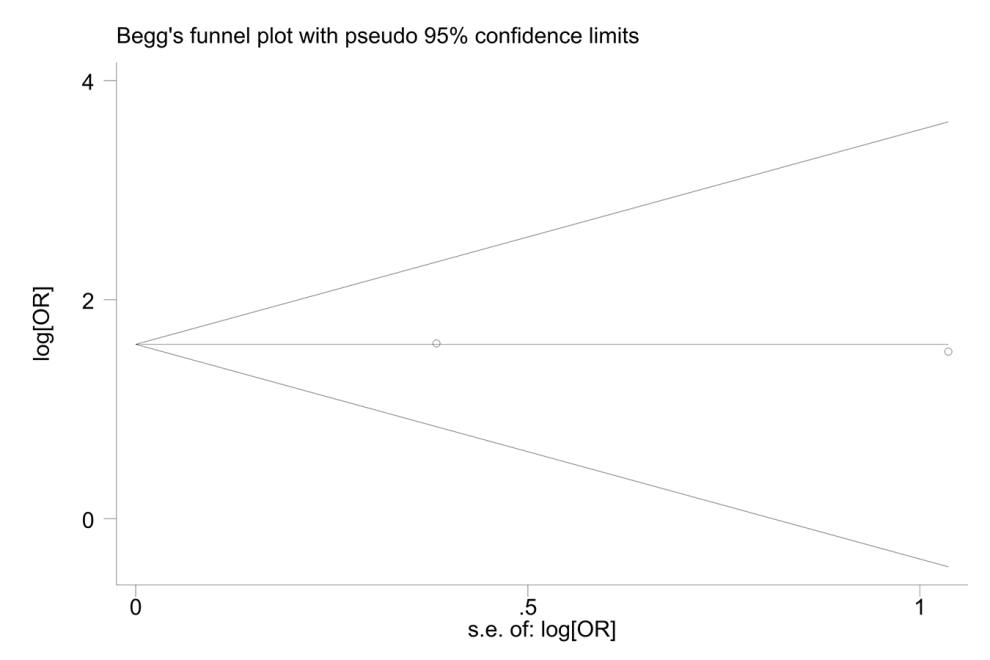
**

**Figure S32** PDA ligation

**
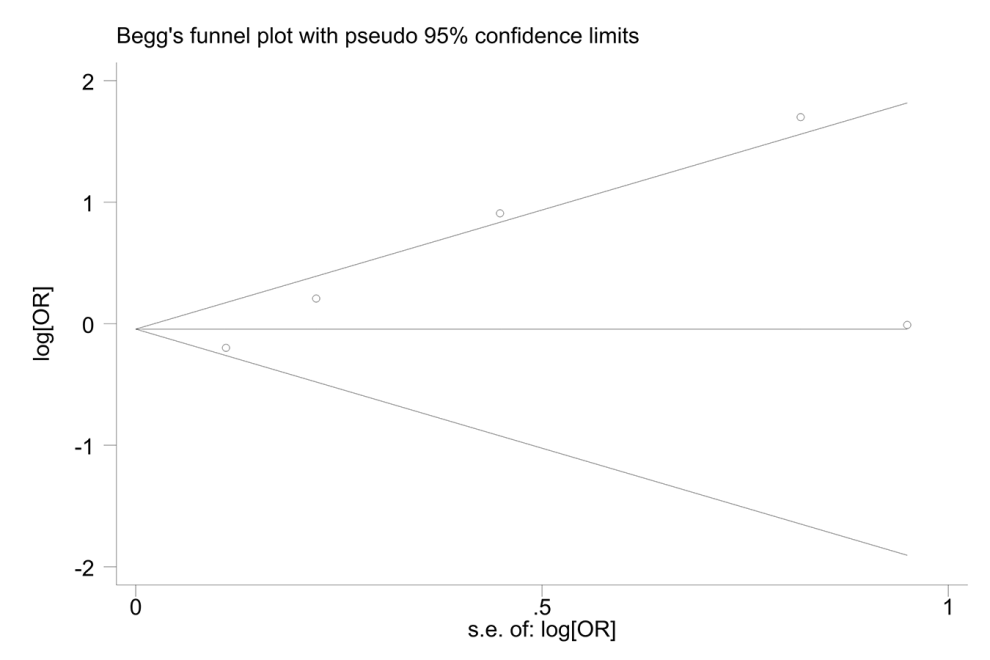
**

**Figure S33** Prematurity/Shorter gestational length

**
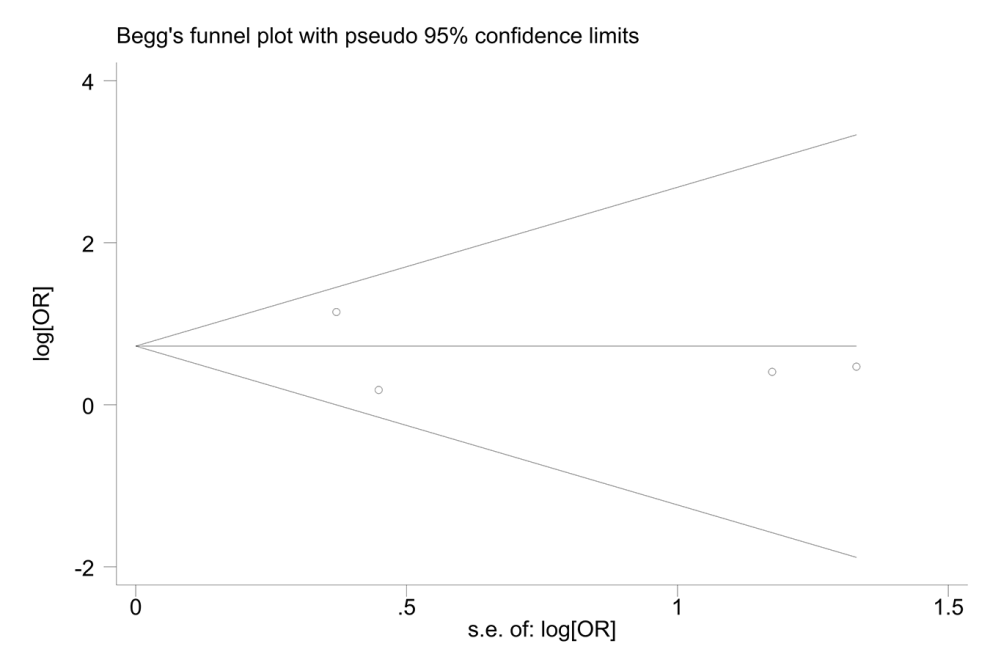
**

**Figure S34** Meningitis

**
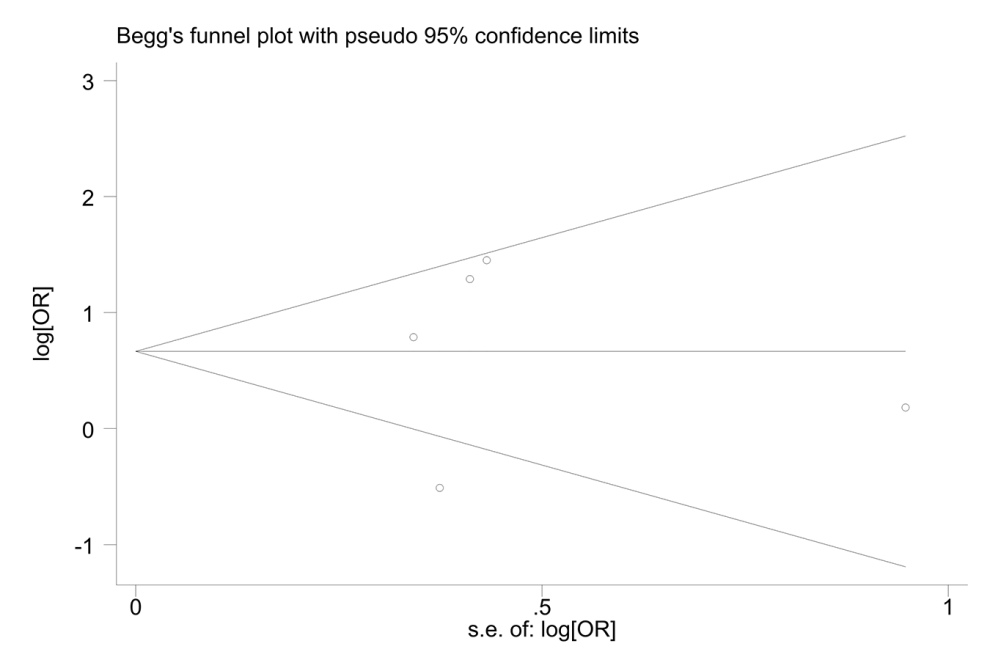
**

**Figure S35** aminoglycoside

**
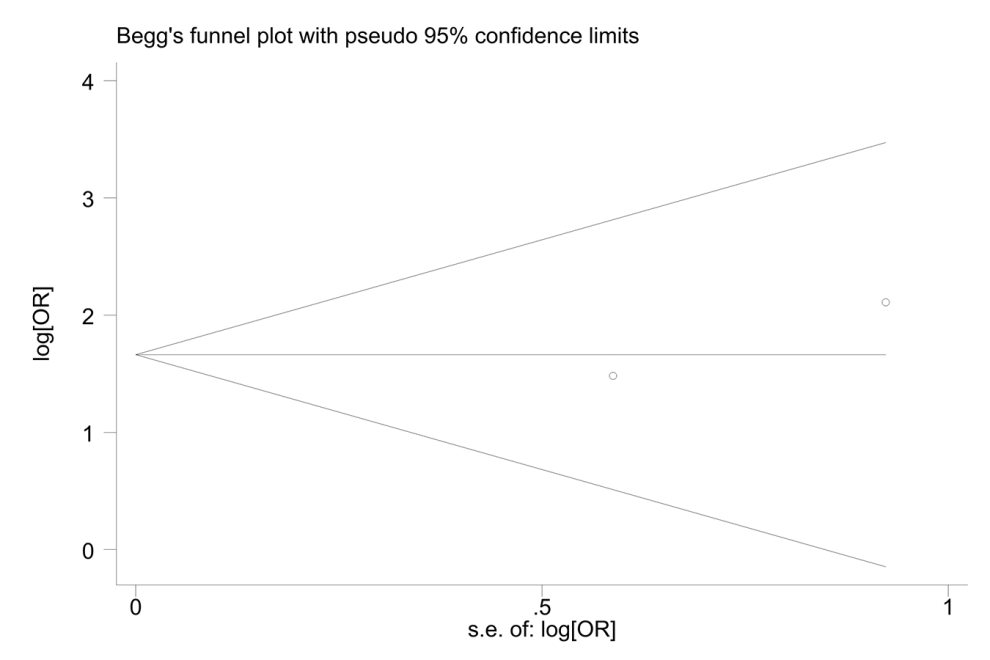
**

**Figure S36** TORCH infection

**
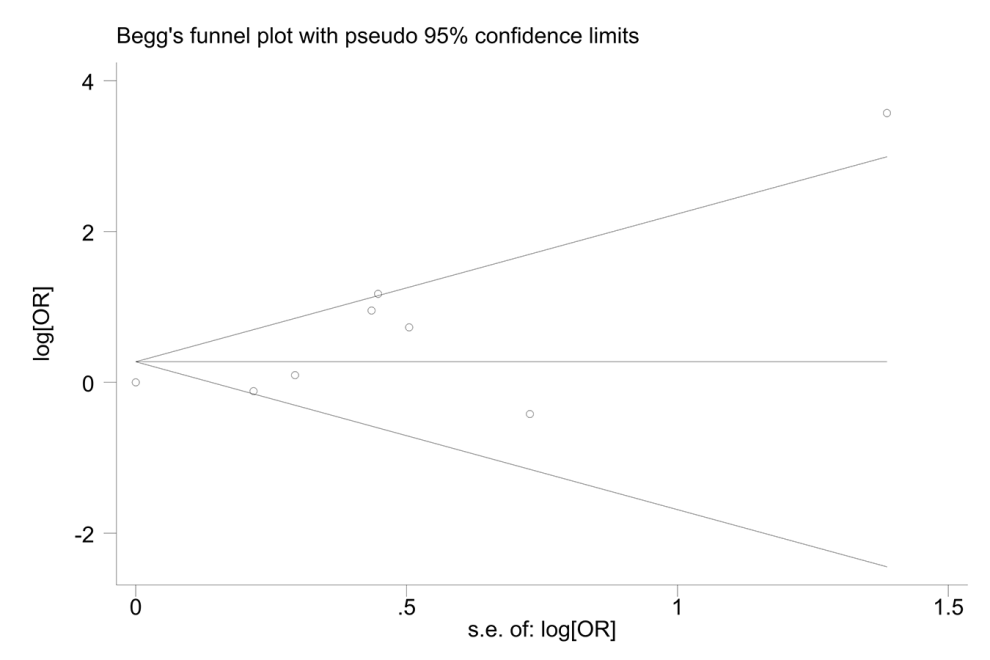
**

**Figure S37** low birth weight（Total）

**
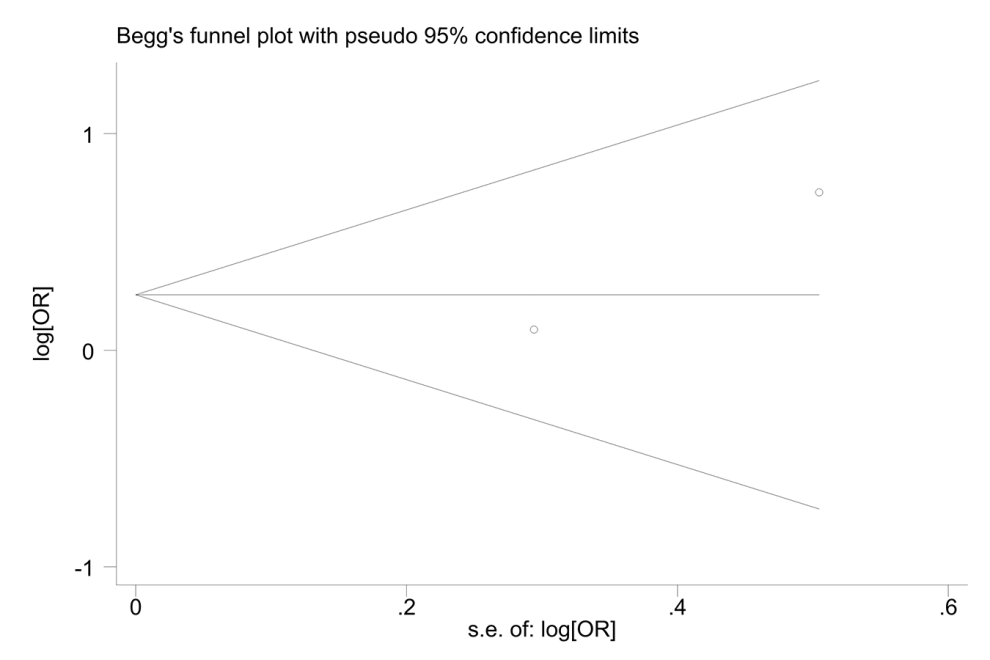
**

**Figure S38** low birth weight（from 1500 to 2500g）

**
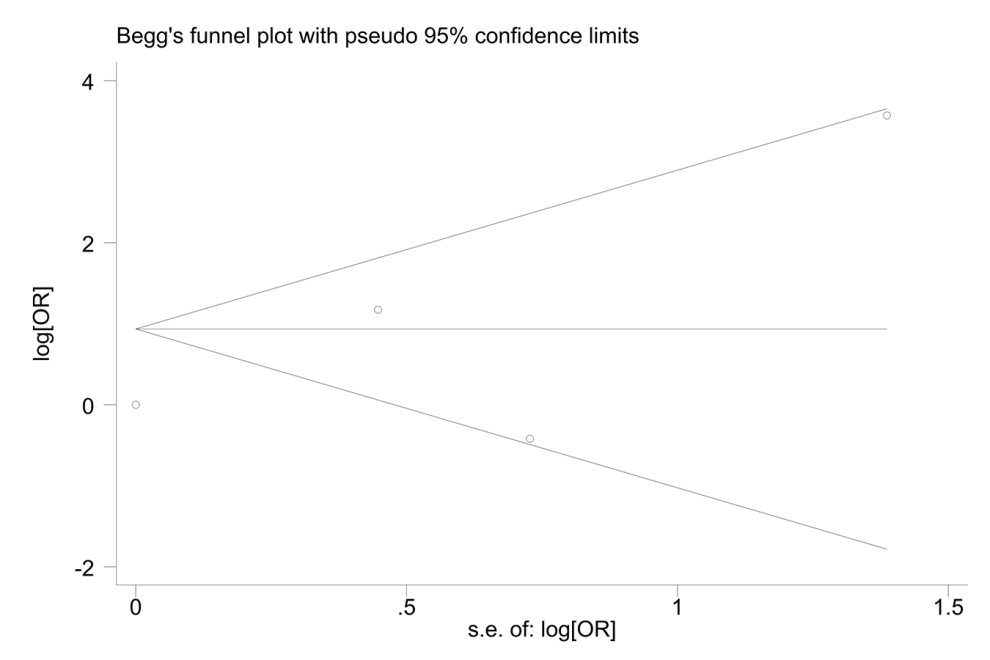
**

**Figure S39** low birth weight（<1500g）

**
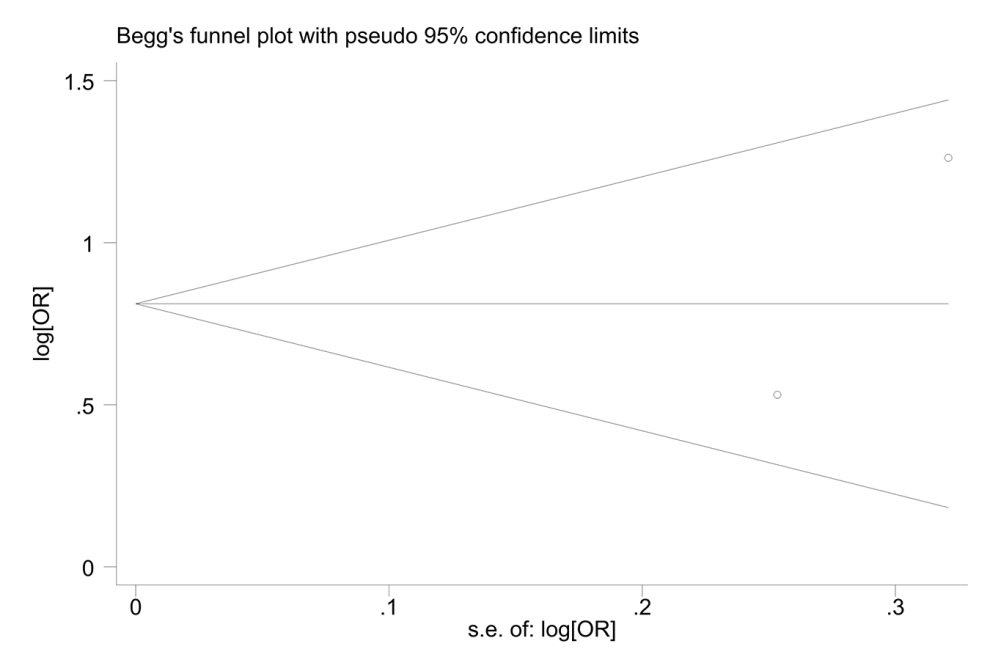
**

**Figure S40** Postnatal hypoxia

**
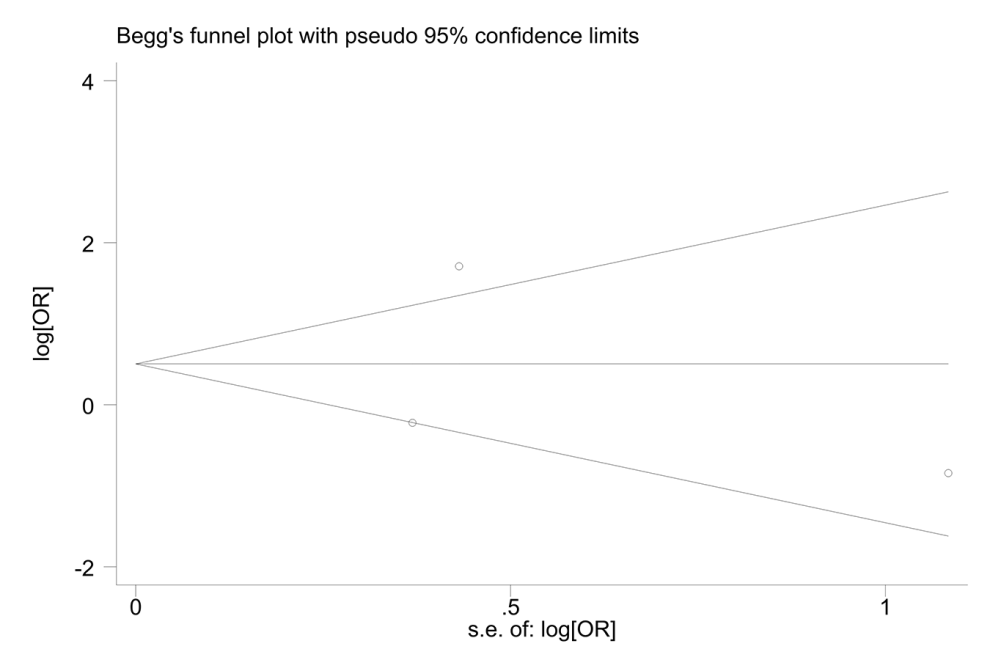
**

**Figure S41** Vancomycin

**
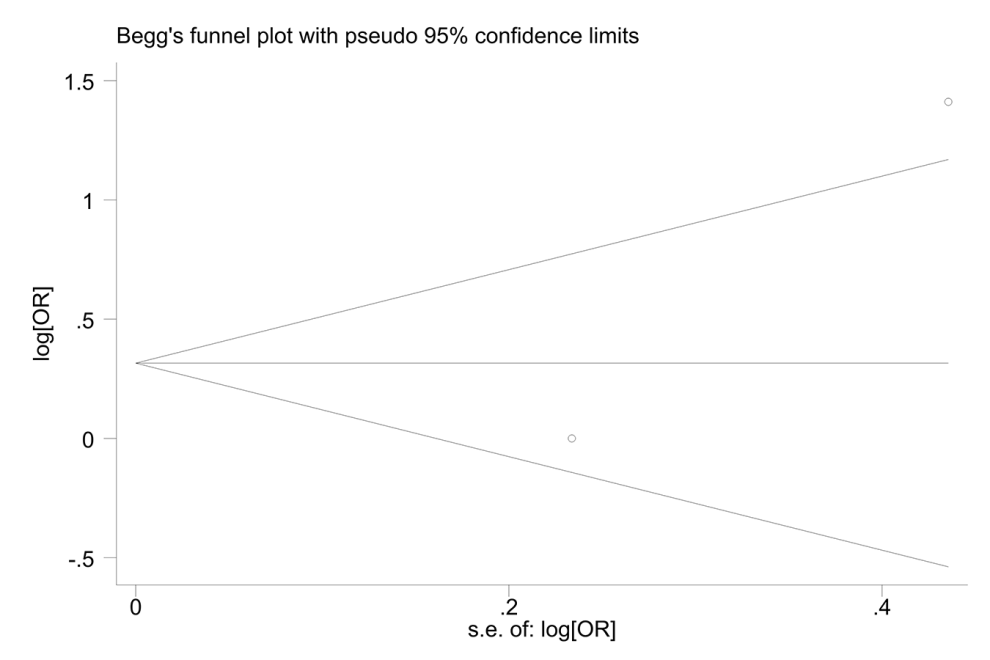
**

**Figure S42** Duration of Vancomycin

**
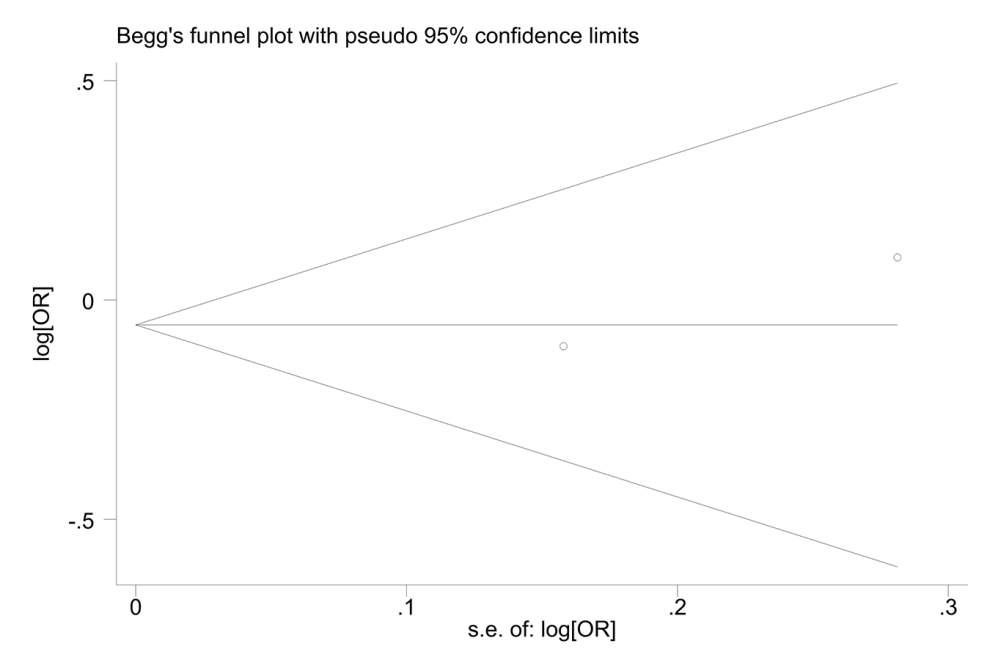
**

**Figure S43** Sex

**
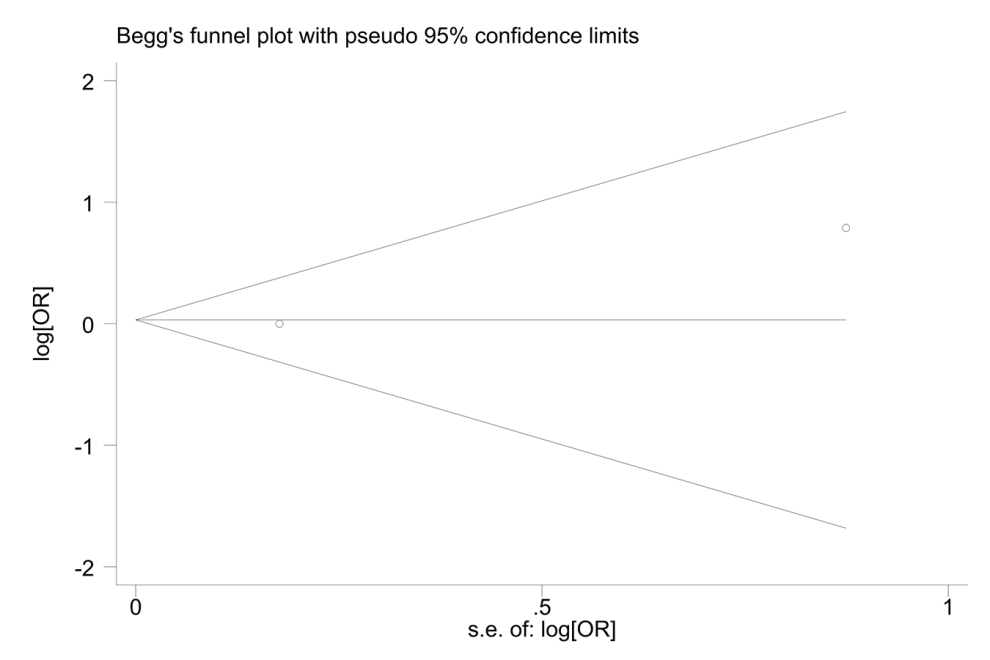
**

**Figure S44** Sepsis
